# Revealing Active Sites and Reaction Pathways in Direct Oxidation of Methane over Fe-Containing CHA Zeolites Affected by the Al Arrangement

**DOI:** 10.1021/jacs.4c11773

**Published:** 2024-11-05

**Authors:** Peipei Xiao, Lizhuo Wang, Hiroto Toyoda, Yong Wang, Kengo Nakamura, Jun Huang, Ryota Osuga, Maiko Nishibori, Hermann Gies, Toshiyuki Yokoi

**Affiliations:** † Institute of Innovative Research, Tokyo Institute of Technology, 4259 Nagatsuta, Midori-ku, Yokohama 226-8501, Japan; ‡ School of Chemical and Biomolecular Engineering, The University of Sydney, Sydney, New South Wales 2006, Australia; § Institute for Catalysis, Hokkaido University, Kita 21 Nishi 10, Kita-ku, Sapporo, Hokkaido 001-0021, Japan; ∥ International Center for Synchrotron Radiation Innovation Smart, Tohoku University, 2-1-1 Katahira, Aoba-ku, Sendai, Miyagi 980-8572, Japan; ⊥ iPEACE223 Inc., Konwa Building, 1-12-22 Tsukiji, Chuo-ku, Tokyo 104-0045, Japan

## Abstract

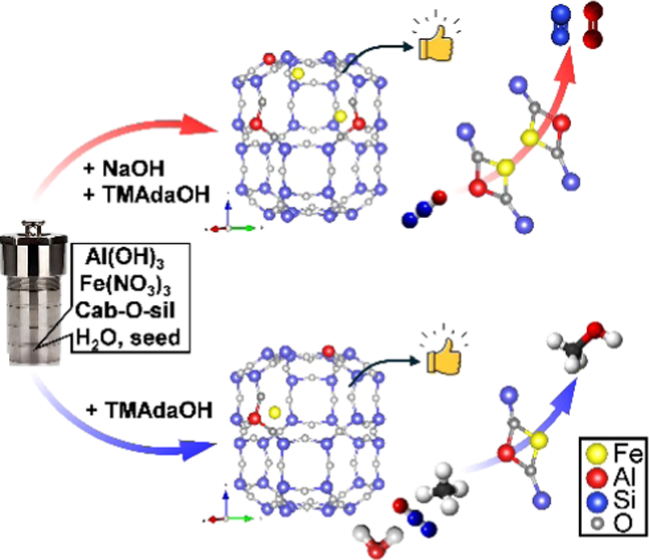

Fe-containing zeolites are effective catalysts in converting the greenhouse gases CH_4_ and N_2_O into valuable chemicals. However, the activities of Fe-containing zeolites in methane conversion and N_2_O decomposition are frequently conflated, and the activities of different Fe species are still controversial. Herein, Fe-containing aluminosilicate CHA zeolites with Fe species at different spatial distances affected by the arrangement of framework Al atoms were synthesized in a one-pot manner in the presence or absence of Na. The arrangement of framework Al atoms was identified by ^27^Al and ^29^Si MAS NMR spectra and thermogravimetry-differential thermal analysis (TG-DTA) curves. Ultraviolet (UV)–vis, X-ray absorption spectroscopy (XAS), and NO adsorption fourier transform infrared spectroscopy (FTIR) spectra were adopted to analyze Fe speciation. The higher proportion of Fe species in the 6 MR of Fe-CHA zeolites in the presence of Na was confirmed by the NO adsorption FTIR spectrum. The activities of proximal and distant Fe sites in reactions including direct oxidation of methane to methanol, methanol to hydrocarbon, and N_2_O decomposition were compared at different temperatures to provide the corresponding active sites and reaction pathways. The distant, isolated Fe and isolated proton were more active in the direct oxidation of methane to methanol and tandem conversion of methanol to hydrocarbon reactions than the proximal, isolated Fe and paired protons, respectively. Additionally, proximal, isolated Fe sites afforded higher activity in N_2_O decomposition. These findings guide the design of highly active catalysts in methane oxidation, methanol to hydrocarbon, and N_2_O decomposition reactions, addressing energy and environmental concerns.

## 1 Introduction

Zeolites are a family of several crystalline microporous materials composed of tetrahedrally coordinated oxygen-bridged Si^4+^ atoms. The substitute of framework Si^4+^ to Al^3+^ makes the tetrahedron [AlO_4_]^−^ anionic lattice charge capable of balancing various extra framework cations, such as protons and metal cations, that serve as the catalytic active sites.^1,2^ The position and amount of Al atoms replacing Si in the framework represent Al distribution and Si/Al ratios.^3^ Distribution and arrangement of framework Al atoms of zeolites are as important as the physical and chemical properties of the zeolite catalysts. Al distribution refers to the spatial position of framework Al atoms in zeolites.^4^ Taking ZSM-5, the aluminosilicate MFI-type three-dimensional (3D) zeolite, as an example, Al atoms are spatially distributed in straight channels, sinusoidal channels, and intersections.^5^ Al arrangement is a description of the distance between the framework Al atoms, being categorized as the proximal Al (Al_p_) and isolated Al (Al_i_).^4^ Specifically, proximal Al (Al_p_) is a system containing two synergistic Al atoms in an arrangement of (Al–O(−Si–O)_*n*=1,2_–Al).^4^ In contrast, the isolated Al (Al_i_) is Al atoms sited far from each other in an arrangement of (Al–O(−Si–O)_*n*>2_–Al).^4^ Both the distribution and arrangement of framework Al atoms have attracted extensive attention and have been widely researched due to the significant effects on the catalytic activity in aspects such as product distribution and lifetime.^4,6^

Chabazite (CHA) zeolites have high-symmetry frameworks comprising single-symmetry T sites. Only one type of isolated framework Al configuration is possible in the CHA zeolite given its single unique T-site.^7^ The proximal framework Al sites are highly dependent on the synthesis conditions. The regulation of framework Al arrangement in CHA zeolite can be realized by altering the cooperation or competition between organic and inorganic structure-directing agents (SDAs),^7−10^ adjusting the starting materials,^11−14^ and choosing different alkali metal cations.^15^ As acidic catalysts, CHA zeolites have been used in methanol to olefin (MTO) and dimethyl ether (DME) to olefin (DTO) reactions.^11,16,17^ On the one hand, the Al arrangement of acidic zeolites influences the product distribution in MTO or DTO reactions.^4,6,18^ Based on computational investigation, Plessow et al. have found that the paired protons arising from Al_p_ take part in the side-chain mechanism of the aromatic cycle, resulting in the formation of ethylene in the MTO process.^19^ In contrast, the isolated proton originating from the Al_i_ contributed to propylene abiding by the paring mechanism.^20^ On the other hand, the Al arrangement of acidic zeolites affects lifetime in MTO or DTO reactions.^4,6,18^ The single proton was reported to be beneficial for the longer lifetime.^10,21^ In addition, CHA zeolites as the excellent carriers are regularly used to load metals such as Fe^13,22^ and Cu.^23−27^ The position and assembly of framework Al atoms directly influence the location and speciation of metal atoms, thus reflecting on the reaction performance such as NH_3_–SCR^22,23,25^ and methane oxidation.^13,24,26,27^

Fe-containing zeolites are active in the direct oxidation of alkanes such as methane, especially utilizing N_2_O or H_2_O_2_ as the oxidant.^28,29^ Due to the excellent activity in methane oxidation and N_2_O decomposition, Fe-containing zeolites have been extensively researched for decades.^29−31^ Sels and co-workers made great efforts on the identification^32,33^ and formation^34,35^ of active Fe species and the influence of the topology structure and Al distribution of zeolites on the activity of Fe species in methane oxidation using N_2_O as the oxidant.^13,36^ The mononuclear α-Fe(II) has been proven by magnetic circular dichroism as the active species, whose exceptional reactivity derives from a constrained coordination geometry enforced by the zeolite lattice.^32^ Nevertheless, reaction results are still lacking to directly compare the performance in methane oxidation between mono- and binuclear Fe sites or distant and proximal Fe sites.

Furthermore, the highly dispersed Fe species with a high Fe content required more framework Al atoms providing negatively charged sites within zeolites to balance the positively charged Fe^3+^ ions.^37,38^ Regularly, the small-pore zeolites such as AEI and CHA can provide more framework Al atoms.^2^ However, Fe-containing zeolite catalysts are generally prepared by postsynthesis methods, namely, ion exchange and wet impregnation.^30,31,33,36,39^ Fe species are prone to aggregate to larger oxide particles by these methods, thus blocking the pores and affecting the reaction activity.^38^ The one-pot synthesis method provided a strategy for the Fe-rich and Al-rich small-pore zeolites with highly dispersed Fe species.^35,37,38^

Herein, we investigated the impact of the framework Al arrangement in CHA zeolite by introducing or eliminating Na^+^ in the synthesis gel on the extra framework Fe species and thus the catalytic activity in the direct oxidation of CH_4_ using N_2_O as the oxidant. The one-pot synthesized Fe-CHA zeolites suppressed cluster formation and enabled even Fe dispersion, which was more reliable for inspecting the impact of framework Al arrangement on Fe species than by the ion-exchange method. A series of spectroscopic data were adopted to analyze the Al arrangement and Fe speciation. The activities of proximal and distant Fe sites affected by framework Al atoms were compared in reactions, including direct oxidation of methane to methanol (DMTM), methanol to hydrocarbon (MTH), and N_2_O decomposition. The active sites and reaction pathways were proposed based on reaction performance.

## 2 Experimental Section

The Fe-containing aluminosilicate CHA-type zeolites (Fe-CHA) were prepared by using *N*,*N*,*N*-trimethyl-1-adamantylammonium hydroxide (TMAdaOH) as OSDA under the hydrothermal conditions based on our previous work.^11^ The Fe-CHA zeolites synthesized in the presence or absence of Na cations were marked as Fe-CHA(Na) and Fe-CHA(Na free), respectively. In a typical synthesis of Fe-CHA(Na), Fe(NO_3_)_3_ solution was mixed and stirred with the alkaline solution containing TMAdaOH, NaOH, and Al(OH)_3_. Afterward, fumed silica (Cab-O-Sil M5) and the seed (SSZ-13 zeolite) were added, and the suspension was stirred fully with the suspension. The prepared gels with the molar ratio of 1 SiO_2_:0.05 Al_2_O_3_:0.2 NaOH:0.2 TMAdaOH:1/2*x* Fe_2_O_3_:30 H_2_O with 5 wt % seed (SSZ-13) (*x* = 50, 100, 400, 800) were crystallized at 150 °C in a rotating oven for 5 days. After being washed, filtered, and dried, the obtained samples were named as-Fe-CHA(Na)-*x*, where *x* means the Si/Fe ratio in the synthesis gel. Subsequently, the as-synthesized products were calcined at 550 °C for 10 h in air to remove OSDA. Afterward, the calcined samples were exchanged twice with a 2.5 M NH_4_NO_3_ aqueous solution at 80 °C for 3 h to get the NH_4_-form samples. Finally, the NH_4_-type samples were calcined at 550 °C for 5 h in air to get H-type Fe-CHA(Na)-*x* zeolites.

The synthesis of Fe-CHA(Na-free) was similar to that of Fe-CHA(Na), just using gels with the molar ratio of 1 SiO_2_:0.05 Al_2_O_3_:0.4 TMAdaOH:1/2*x* Fe_2_O_3_:30 H_2_O with 5 wt % seed to crystallize at 150 °C in a rotating condition for 5 days. The final obtained samples were named Fe-CHA(Na free)-*x*, where *x* means the Si/Fe ratio in the synthesis gel.

For comparison, the aluminosilicate CHA zeolites were synthesized via a procedure similar to Fe-CHA(Na) and Fe-CHA(Na free) without an Fe source in the synthesis gel, and the obtained products were nominated as CHA(Na) and CHA(Na-free), respectively. The NH_4_-type CHA zeolites were exchanged with 2 mmol·L^–1^ Fe(NO_3_)_3_ solution at 80 °C stirring for 24 h. After filtering, washing, drying, and calcination in air at 550 °C for 5 h, the obtained samples were denoted as IE-Fe/CHA(Na) and IE-Fe/CHA(Na-free).

The details of the catalyst characterization and catalytic activity test are comparable to our previous work^38^ and are documented in the Supporting Information.

## 3 Results and Discussion

### 3.1 Catalyst Characterization

#### 3.1.1 Physicochemical Properties

**Figure 1:**
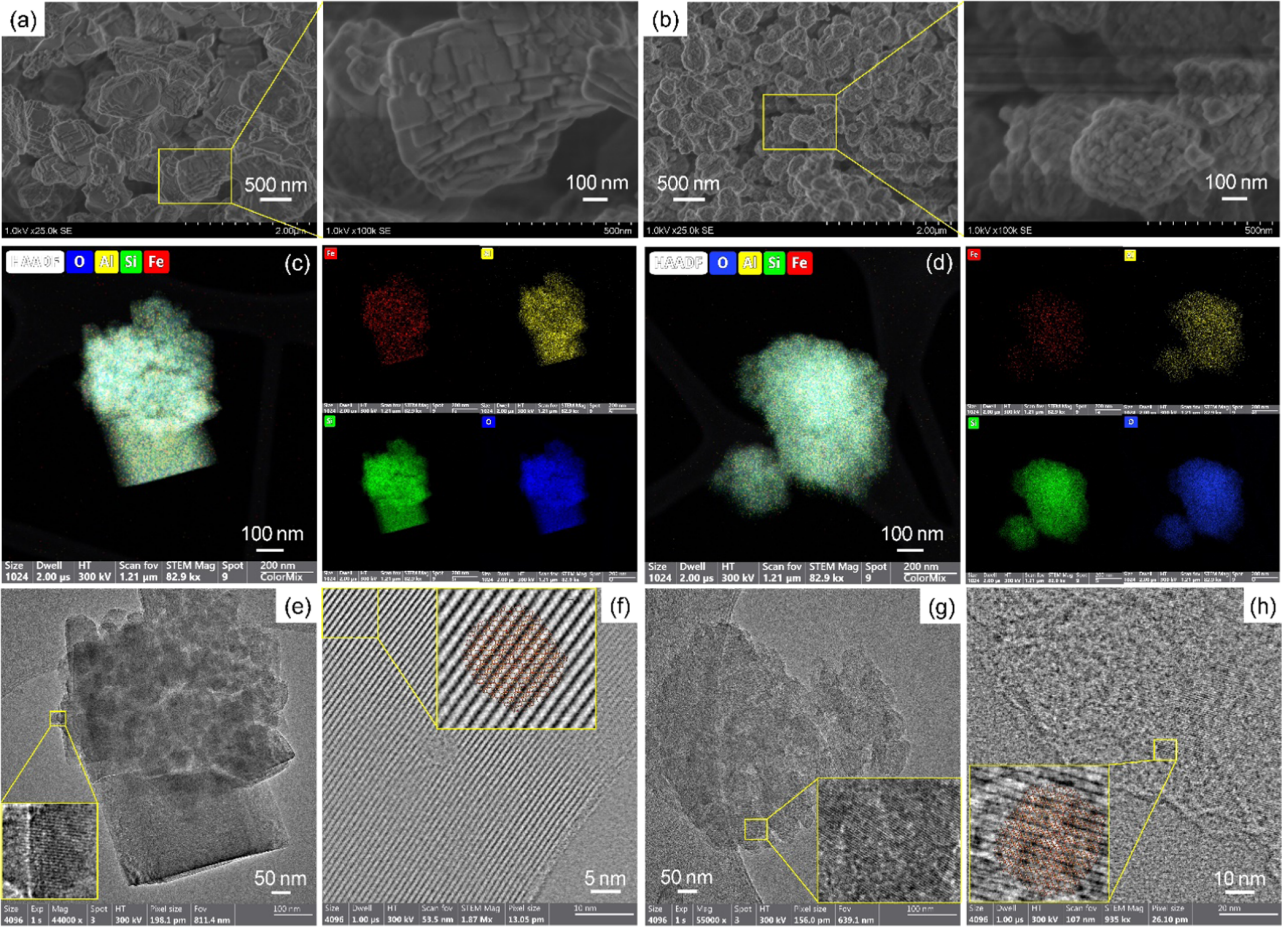
SEM images of (a) Fe-CHA(Na)-100 and (b) Fe-CHA(Na-free)-100. HAADF-STEM images and corresponding EDS element mapping images of (c) Fe-CHA(Na)-100 and (d) Fe-CHA(Na free)-100. HRTEM images of (e) Fe-CHA(Na)-100 and (g) Fe-CHA(Na free)-100. iDPC-STEM images of (f) Fe-CHA(Na)-100 and (h) Fe-CHA(Na-free)-100.

The X-ray diffraction (XRD) patterns of the as-synthesized Fe-CHA samples verified the CHA topology structure with a series of characteristic peaks at 9.6, 15.9, 20.8, 25.0, 26.4, and 31.2° (Figure S1).^40^ No features belonging to iron oxide were observed, suggesting highly dispersed Fe species in Fe-CHA zeolites. The chemical composition demonstrated the analogous Al and Fe content between Fe-CHA(Na) and Fe-CHA(Na free) zeolite catalysts in Table 1. The OSDA contents in Fe-CHA(Na) and Fe-CHA(Na-free) were around 19 and 22%, respectively (Figure S2). The exothermic peak of as-Fe-CHA(Na-free) at around 425 °C was lower than that of as-Fe-CHA(Na) at around 436 °C, possibly indicating that OSDA in Fe-CHA(Na free) was mainly located in a wider space such as eight-membered rings (8 MR) (Figure S2).^41,42^ Correspondingly, the OSDA in Fe-CHA(Na) was most likely placed in a narrow space (Figure S2). Moreover, it has been reported that OSDA with Na was prone to guide Al pairs in 6 MR.^7^ The SEM images presented an irregular shape with small particles covered on the surface (Figures 1a,b and S3). Fe-CHA(Na)-50 and Fe-CHA(Na)-100 displayed particle sizes larger than those of Fe-CHA(Na free)-50 and Fe-CHA(Na free)-100, respectively. Additionally, the size of small particles on the surfaces of Fe-CHA(Na)-50 and Fe-CHA(Na)-100 was larger than that of Fe-CHA(Na-free)-50 and Fe-CHA(Na-free)-100, respectively. The smaller particles on the surface of Fe-CHA(Na-free) zeolites contributed to the stacked holes and thus the higher total volume (Table S1). The result was validated by the strong uptake at 0.95 < *p*/*p*_0_ < 1.0 in the N_2_ adsorption and desorption isotherms (Figure S4). Moreover, the strong uptake at low *p*/*p*_0_ revealed the presence of micropores (Figure S4 and Table S1). The Brunauer–Emmett–Teller (BET) surface area and external surface area of Fe-CHA(Na) were slightly lower than those of Fe-CHA(Na-free); meanwhile, both the BET surface area and external surface area decreased with the Fe content growth (Table S1). The high-angle annular dark-field scanning transmission electron microscopy (HAADF-STEM) images, corresponding energy dispersive spectroscopy (EDS) elemental mapping images, and high resolution TEM (HRTEM) images for Fe-CHA(Na) and Fe-CHA(Na-free) exhibited uniformly dispersed Fe species (Figures 1c,d and S5). The HRTEM images established that small particles accumulated on the surface of large particles for both Fe-CHA(Na) and Fe-CHA(Na-free) (Figure 1e,g). The iDPC-STEM images are displayed from the [010] projection, which aligned well with the CHA structure (Figures 1f,h and S5).

**Table 1:** Chemical Composition and Acid Amount of the Fe-CHA Zeolites

	chemical compositions^a^				acid amount (mmol/g)^b^			
sample	Si/Al	Si/Fe	Fe/Al	Fe (wt %)	weak	medium	strong	total
Fe-CHA(Na)-800	11.5	1520	0.007	0.05	0.51	0.26	0.52	1.29
Fe-CHA(Na)-400	11.6	680	0.017	0.11	0.53	0.28	0.53	1.34
Fe-CHA(Na)-100	12.2	151	0.081	0.54	0.59	0.45	0.52	1.56
Fe-CHA(Na)-50	11.7	61	0.192	1.31	0.50	0.39	0.79	1.68
Fe-CHA(Na free)-800	12.9	1120	0.011	0.07	0.38	0.19	0.49	1.06
Fe-CHA(Na free)-400	12.7	520	0.024	0.16	0.42	0.25	0.55	1.22
Fe-CHA(Na free)-100	12.6	122	0.103	0.67	0.35	0.27	0.57	1.19
Fe-CHA(Na free)-50	12.8	68	0.188	1.19	0.44	0.53	0.89	1.86

a Determined by ICP-AES.

b Determined by NH_3_-TPD; the weak, medium, and strong acid amounts were fitted at approximately 150, 300, and 400–450 °C, respectively.

#### 3.1.2 Al Arrangement

**Figure 2:**
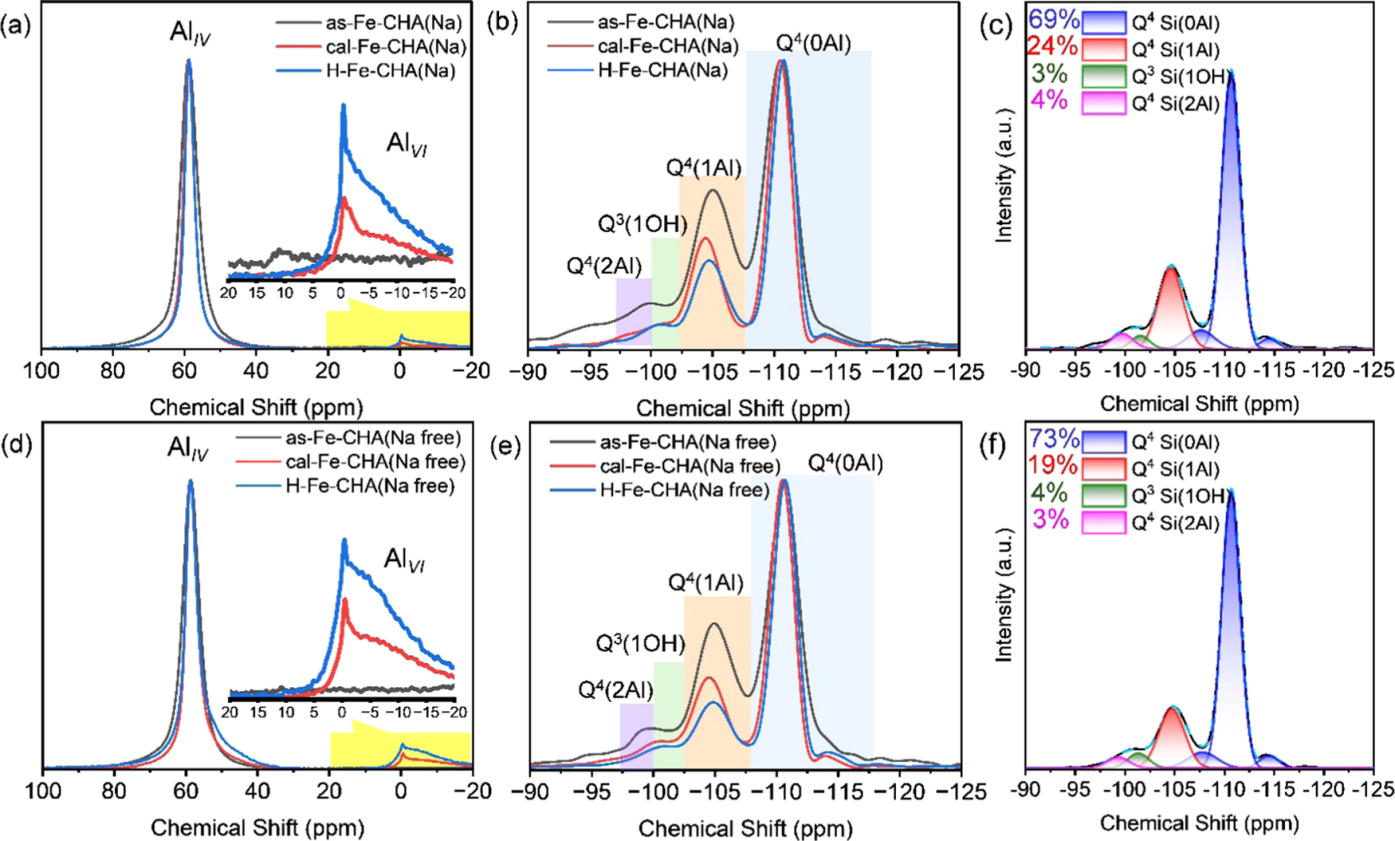
^27^Al MAS NMR spectra for the as-synthesized, calcined, and H-type (a) Fe-CHA(Na)-100 and (d) Fe-CHA(Na free)-100. ^29^Si MAS NMR spectra for the as-synthesized, calcined, and H-type (b) Fe-CHA(Na)-100 and (e) Fe-CHA(Na free)-100. Deconvolution of ^29^Si MAS NMR spectra for H-type (c) Fe-CHA(Na)-100 and (f) Fe-CHA(Na free)-100.

Solid-state ^27^Al and ^29^Si MAS NMR techniques have been adopted to analyze the framework environment of Fe-CHA zeolites (Figures 2 and S6–S8). The ^27^Al MAS NMR spectra of the as-synthesized, calcined, and H-type Fe-CHA zeolites with the Si/Fe ratio of 100 in the synthesis gel uncovered the intense peak at ca. 58 ppm assigned to the tetra-coordinated Al (Al_IV_) in the framework and the weak peak at ca. 0 ppm attributed to the hexa-coordinated Al (Al_VI_) on the extra framework (Figure 2a,d).^28,29^ Affected by Fe in the framework, the peak of Al_IV_ for as-Fe-CHA was wider than the calcined and H-type ones since a part of the framework Fe and Al migrated to the extra framework after calcination. In addition, H-type Fe-CHA zeolites with different Si/Fe ratios displayed the main Al_IV_ peaks at 58 ppm and weak Al_VI_ peaks at 0 ppm (Figure S6a,b). The ^29^Si MAS NMR spectra of the as-synthesized, calcined, and H-type Fe-CHA zeolites were used to expose the environment of Si (Figures 2b,e and S6–S8). Due to the interference of Fe, the ^29^Si CPMAS NMR spectra of H-type Fe-CHA zeolites did not exhibit any peak (Figure S7a). However, the peaks of Q^3^(Si(OSi)_3_OH) at −100 ppm were observed on CHA zeolites without Fe (Figure S7b). The intensity of bands belonging to Q^4^(1Al) and Q^4^(2Al) decreased after calcination due to dealumination (Figure 2b,e).^43^ Representatively, the ^29^Si MAS NMR spectra of H-type Fe-CHA(Na) and Fe-CHA(Na free) were deconvoluted and the proportion of different Si environments has been calculated based on the peak area (Figure S8 and Table S2). A higher proportion of Q^4^(2Al) and Q^4^(1Al) for Fe-CHA(Na) was obtained than that of Fe-CHA(Na-free) (Figure 2c,f). Q^4^(2Al) represented Al–O–(Si–O)–Al or the second nearest neighbor (2NN), one kind of paired Al configuration. In addition, Q^4^(1Al) corresponded to Al–O–(Si–O)_3_–Al and Al–O–(Si–O)_2_–Al; among them, Al–O–(Si–O)_2_–Al or the third nearest neighbor (3NN) symbolized another kind of paired Al configuration.^44^ Thus, we concluded that Fe-CHA(Na) obtained more Al_p_ than Fe-CHA(Na free) based on ^29^Si MAS NMR spectra.

#### 3.1.3 Fe Speciation

**Figure 3:**
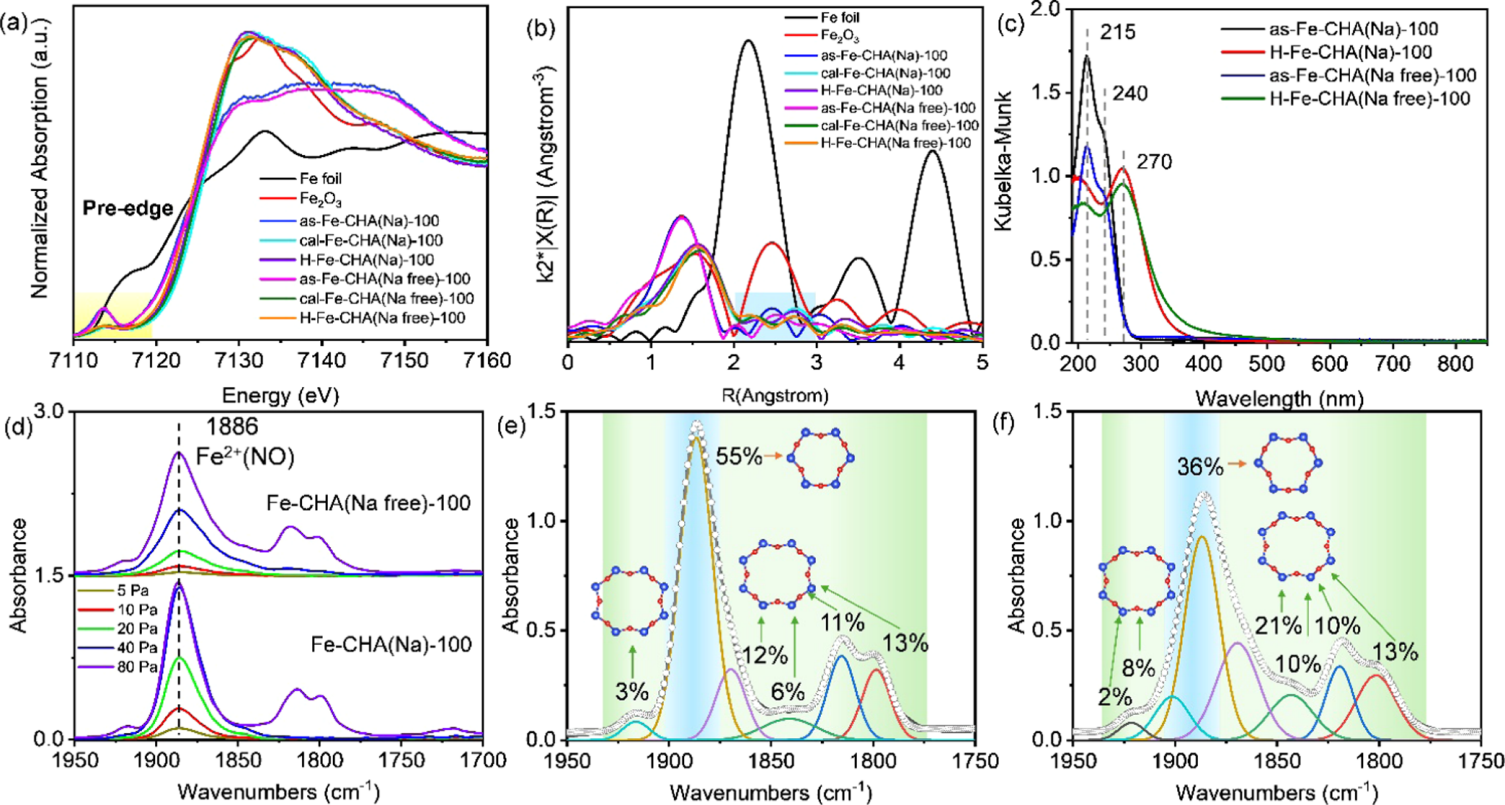
(a) Normalized XANES spectra and (b) Fourier-transformed EXAFS spectra at the Fe K-edge for the as-synthesized, calcined, and H-type Fe-CHA(Na) and Fe-CHA(Na free) zeolite catalysts, Fe foil, and Fe_2_O_3_ as references. (c) UV–vis spectra of the as-synthesized and H-type Fe-CHA(Na) and Fe-CHA(Na free). (d) NO adsorption FTIR spectra (*P*_NO_ = 5–80 Pa) of Fe-CHA(Na)-100 and Fe-CHA(Na free)-100 zeolite catalysts at −120 °C after evacuation at 500 °C for 1 h. Deconvolution of NO adsorption (*P*_NO_ = 80 Pa) FTIR spectra for H-type (e) Fe-CHA(Na) and (f) Fe-CHA(Na free).

X-ray absorption spectra (XAS) of the as-made, calcined, and H-type Fe-CHA(Na) and Fe-CHA(Na free) zeolites were measured. The normalized Fe K-edge X-ray absorption near-edge structure (XANES) and Fourier-transformed extended X-ray absorption fine structure (FT-EXAFS) spectra of the as-made, calcined, and H-type Fe-CHA(Na) and Fe-CHA(Na free) samples are demonstrated in Figure 3a,b.^38,45^ The symmetry of the pre-edge is an indicator of octahedral and tetrahedral environments with Fe species. The stronger and symmetric pre-edge for the as-made samples indicated the tetrahedral coordination of the Fe species. The weaker and asymmetric pre-edges for calcined and H-type samples indicated the tetrahedral and octahedral coordination Fe species. The Fourier-transformed EXAFS spectra provided information on the radial distance between Fe and other elements (Figure 3b).^45^ A shorter radial distance at 1.4 Å of the as-made samples was provided than 1.6 Å of calcined and H-type samples due to the framework Fe of the as-made samples. The main Fe component of both Fe-CHA(Na) and Fe-CHA(Na free) zeolites was the first shell Fe–O at 1–2 Å, which meant that the Fe species existed as isolated Fe. The intensity of the second shell Fe–O and Fe–Fe at 2–3 Å was weak. Therefore, mentioning the proximal Fe species rather than Fe pairs and distant Fe species rather than isolated Fe species is more accurate. Reasonable EXAFS fitting parameters were difficult to obtain due to the quite low Fe/Al ratio and density (Table S3).

Moreover, the coordination state and extent of aggregation of Fe species in the as-synthesized and H-type Fe-CHA zeolites were investigated by UV–vis spectroscopy (Figures 3c and S9). The spectra of the as-synthesized zeolites were dominated by two characteristic oxygen-to-metal charge-transfer bands around 215 and 240 nm, which were related to tetrahedral Fe^3+^ species in the framework (Figure 3c).^38,46^ The result was aligned with the XANES spectra in Figure 3a. Thus, the conclusion that Fe^3+^ was predominantly built into the zeolite framework via the Fe^3+^ isomorphous substitution of Si^4+^ was made. Compared to the as-synthesized zeolites, the band at 240 nm shifted to 270 nm for H-type samples, which was typical for octahedral Fe species (Figure 3c).^46^ The result was consistent with the XANES spectra in Figure 3a. The noticeable difference between Fe-CHA(Na) and Fe-CHA(Na free) with various Si/Fe ratios was not observed.

The NO adsorption FTIR spectroscopy was utilized to access the speciation and location of Fe species at −120 °C after activation at 500 °C for 1 h. As exposed in Figure 3d, Fe-CHA zeolites obtained the main band at 1886 cm^–1^ belonging to Fe^2+^NO located in 6 MR at low NO pressure, the shoulder band at 1852 cm^–1^ belonging to Fe^2+^(NO)_2_ positioned in 8 MR at medium NO pressure, and the bands at 1801, 1810, and 1917 cm^–1^ belonging to Fe^2+^(NO)_3_ sited in 8 MR at high NO pressure.^47^ Note that Fe^2+^ originated from Fe^3+^ “self-reduction” under vacuum conditions.^47^ Fe-CHA(Na) and Fe-CHA(Na free) showed 55 and 36% of the peak area at 1886 cm^–1^, respectively, by deconvolution of the NO adsorption FTIR spectra when the NO pressure was 80 Pa (Figure 3e,f). The finding revealed that Fe species in Fe-CHA(Na free) and Fe-CHA(Na) were primarily located in 8 and 6 MR, respectively.^47^ The location of Fe species provided convincing evidence that the Al distribution influenced Fe species. What is more is that the location of Fe species affected by Al distribution was highly consistent with the TG-DTA curves that Al atoms were mainly sited in 8 MR for Fe-CHA(Na free) influenced by the size of OSDA (Figure S2). It is worth pointing out that the proportion of Fe species in 6 MR for cal-Fe-CHA(Na) was decreased compared with that of H-type Fe-CHA(Na) (Figure S9e–g). The existence of Na inhibited the formation of Fe sites on the extra framework. The phenomenon was in line with the conclusion that OSDA or Na can be in 6 MR.^7^

#### 3.1.4 Acidic Properties

**Figure 4:**
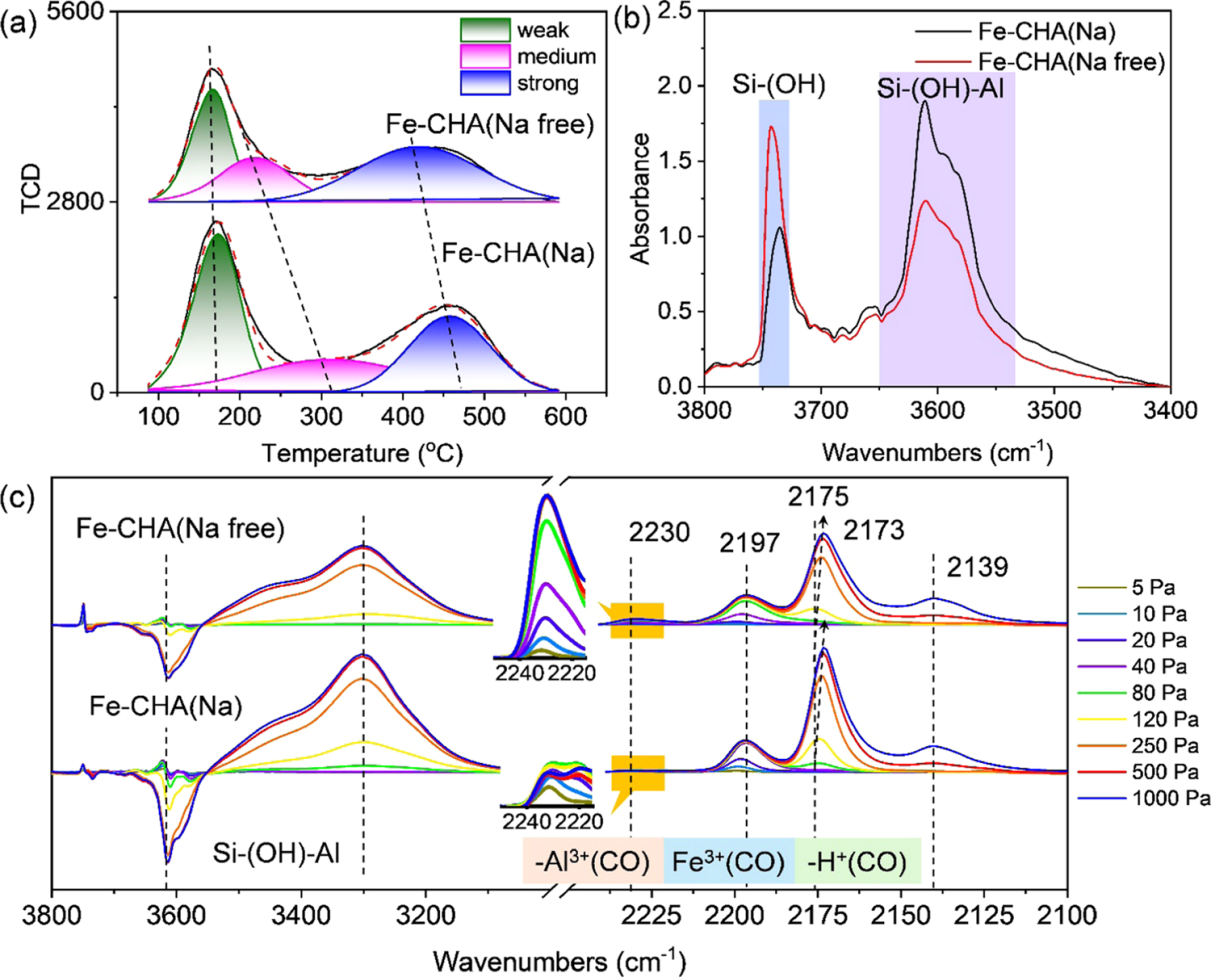
(a) NH_3_-TPD, (b) hydroxyl vibration, and (c) CO adsorption FTIR spectra (*P*_CO_ = 5–1000 Pa) at −120 °C after being evacuated at 500 °C for 1 h for Fe-CHA(Na) and Fe-CHA(Na free) zeolite catalysts with the Si/Fe ratio of 100 in the synthesis gel.

The acid content of Fe-CHA zeolites was first analyzed by NH_3_-TPD (Figures 4a and S10). The NH_3_-TPD spectra were deconvoluted into three peaks and classified as weak, medium, and strong acid sites originating from the physically adsorbed NH_3_ and weak Lewis acid sites (LAS) and weak Brønsted acid sites (BAS) (Si–OH–Al), respectively.^38^Table 1 displays the acid amount estimated from the fitting peak area of NH_3_-TPD profiles. Except for Fe-CHA(Na free)-50, Fe-CHA(Na) contained more acid than Fe-CHA(Na free), ignoring the Fe content due to the slightly lower Si/Al ratio of Fe-CHA(Na). In addition, the temperature corresponding to the center of the fitting peak of Fe-CHA(Na) was higher than that of Fe-CHA(Na free) (Figure 4a), indicating the weaker acid strength of Fe-CHA(Na free) possibly due to the location of Al in 8 MR.^48,49^

The FTIR spectra in the hydroxyl vibration region for Fe-CHA zeolites are illustrated in Figure 4b. The prominent hydroxyl band at 3616 cm^–1^ with a shoulder at 3585 cm^–1^ corresponded to the Brønsted hydroxyl groups.^47^ It has been signified that the intensity of Brønsted hydroxyl groups for Fe-CHA(Na) was stronger than that of Fe-CHA(Na free), in agreement with the NH_3_-TPD results. The bands at 3745 and 3735 cm^–1^ were assigned to the terminal silanol groups on the external surface of zeolites.^47^ The intensity of the silanol band for Fe-CHA(Na free) was stronger than that of Fe-CHA(Na) due to the smaller particle size of Fe-CHA(Na free) zeolites (Figure 1).

CO adsorption FTIR technique was further used to analyze the types of acid sites of Fe-CHA zeolites at −120 °C after activation at 500 °C for 1 h (Figure 4c). The low-frequency feature at 2197 cm^–1^ was observed at low CO pressure and shifted to 2194 cm^–1^ along with the CO pressure increasing, assigned to the CO molecules adsorbed on Fe^2+^ ions.^38,47^ With the CO pressure further increasing, the shoulder band at 2175 cm^–1^ appeared and gradually shifted to 2173 cm^–1^, which was attributed to the ν_C–O_ vibration of the adsorbed CO interacting with zeolitic OH groups via weak hydrogen bonds.^34^ The weak band at 2230 cm^–1^ was attributed to the stretching mode of ν(C≡O) on LAS.^5^ Its intensity of Fe-CHA(Na free) was stronger than that of Fe-CHA(Na), indicating more LAS of Fe-CHA(Na free). The broad FTIR feature centered at around 3300 cm^–1^ and the negative features in the vibrational region of zeolitic OH groups at 3616 cm^–1^ were observed. The stronger intensity of the band at 3300 cm^–1^ for Fe-CHA(Na) agreed with the NH_3_-TPD results in Figure 4a.

### 3.2 Reaction Performance

#### 3.2.1 Oxidation of Methane to Methanol

**Figure 5:**
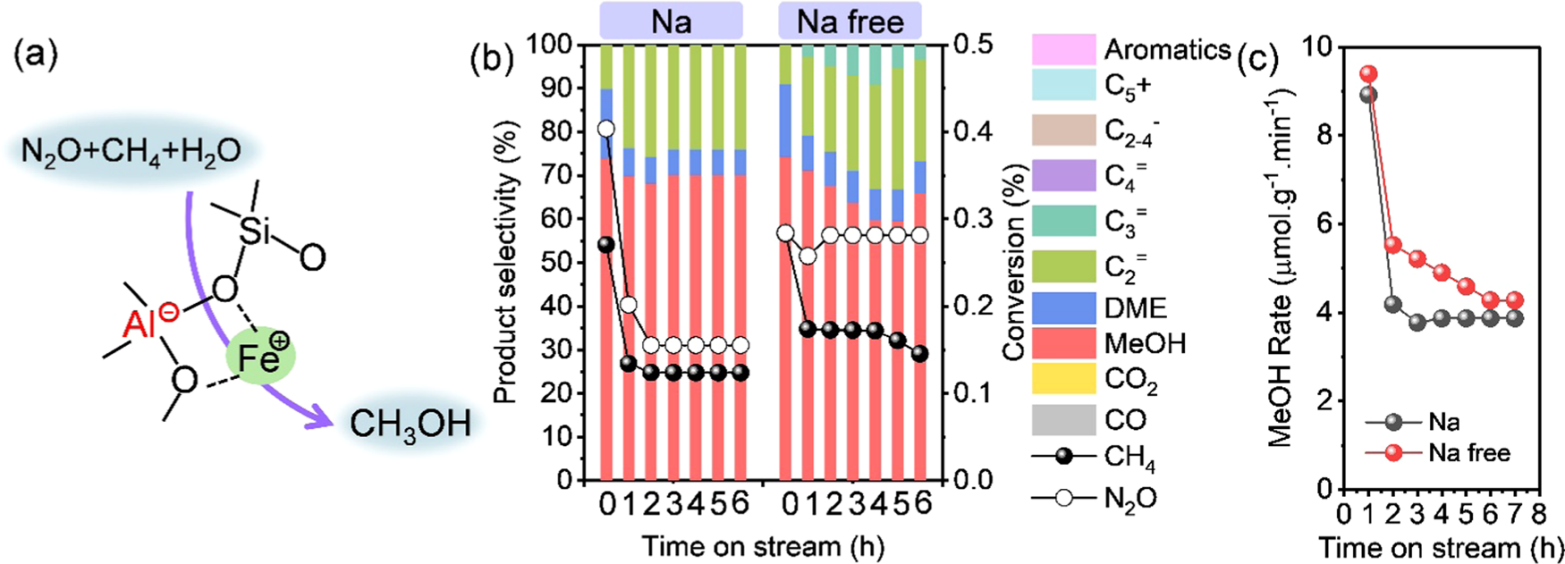
(a) Direct oxidation of methane to methanol on active Fe sites over Fe-containing zeolites. Comparing (b) conversion and selectivity and (c) methanol formation rate of H-type Fe-CHA(Na)-100 and Fe-CHA(Na free)-100 at 250 °C. Reaction conditions: 100 mg of catalyst, CH_4_/N_2_O/H_2_O/Ar = 10/10/2/3 mL·min^–1^, and WHSV = 15,000 mL·g^–1^·h^–1^.

In our previous report, high methanol selectivity was obtained at 250 °C for Fe-AEI zeolite catalysts with neither the tandem reaction of MTH nor N_2_O decomposition.^38^ Therefore, the reaction was executed at 250 °C or lower to investigate the activity of Fe species in the oxidation of methane to methanol in this study (Figure 5a). Representatively, Fe-CHA(Na)-100 and Fe-CHA(Na free)-100 were used. As displayed in Figure 5b, around 70% methanol selectivity was obtained with Fe-CHA(Na)-100 and Fe-CHA(Na free)-100. Both the methane conversion and methanol formation rate of Fe-CHA(Na free)-100 were higher than those of Fe-CHA(Na)-100, indicating the more active Fe species or higher activity of Fe species in Fe-CHA(Na free) (Figure 5b,c). It was worth mentioning that IE-Fe/CHA(Na free) zeolite catalysts (Figures S11–S18 and Table S4) also obtained higher methane conversion and higher methanol formation rate with more than 95% (MeOH + DME) selectivity at 250 °C than IE-Fe/CHA(Na) (Figure S19). Because Fe-CHA(Na free) and IE-Fe/CHA(Na free) contributed to the distant, isolated Fe sites, they were influenced by Al_*i*_. Distant Fe sites were concluded to show higher activity in the DMTM reaction than proximal Fe sites affected by Al_p_.

The comparison of activities in methane oxidation over proximal and distant Fe sites has always been a hot topic. The initial research for methane oxidation using H_2_O_2_ was based on a binuclear Fe species provided by Hutchings and co-workers.^50,51^ However, in recent years, the reports have claimed that mononuclear Fe has higher activity than binuclear ones.^31,37,52,53^ It is important to note that these studies were limited to alkane oxidation in the liquid phase using H_2_O_2_ as an oxidant. Recently, Zhao et al. have reported that binuclear Fe sites over Fe-FER-SSIE exhibited better stability, resulting in higher methane conversion and selectivity to methanol and DME using N_2_O as the oxidant.^39^ However, the same research on Fe-CHA zeolites from Bols et al. was established on the mononuclear Fe site.^35^

#### 3.2.2 Tandem Oxidation of Methane to Hydrocarbon

**Figure 6:**
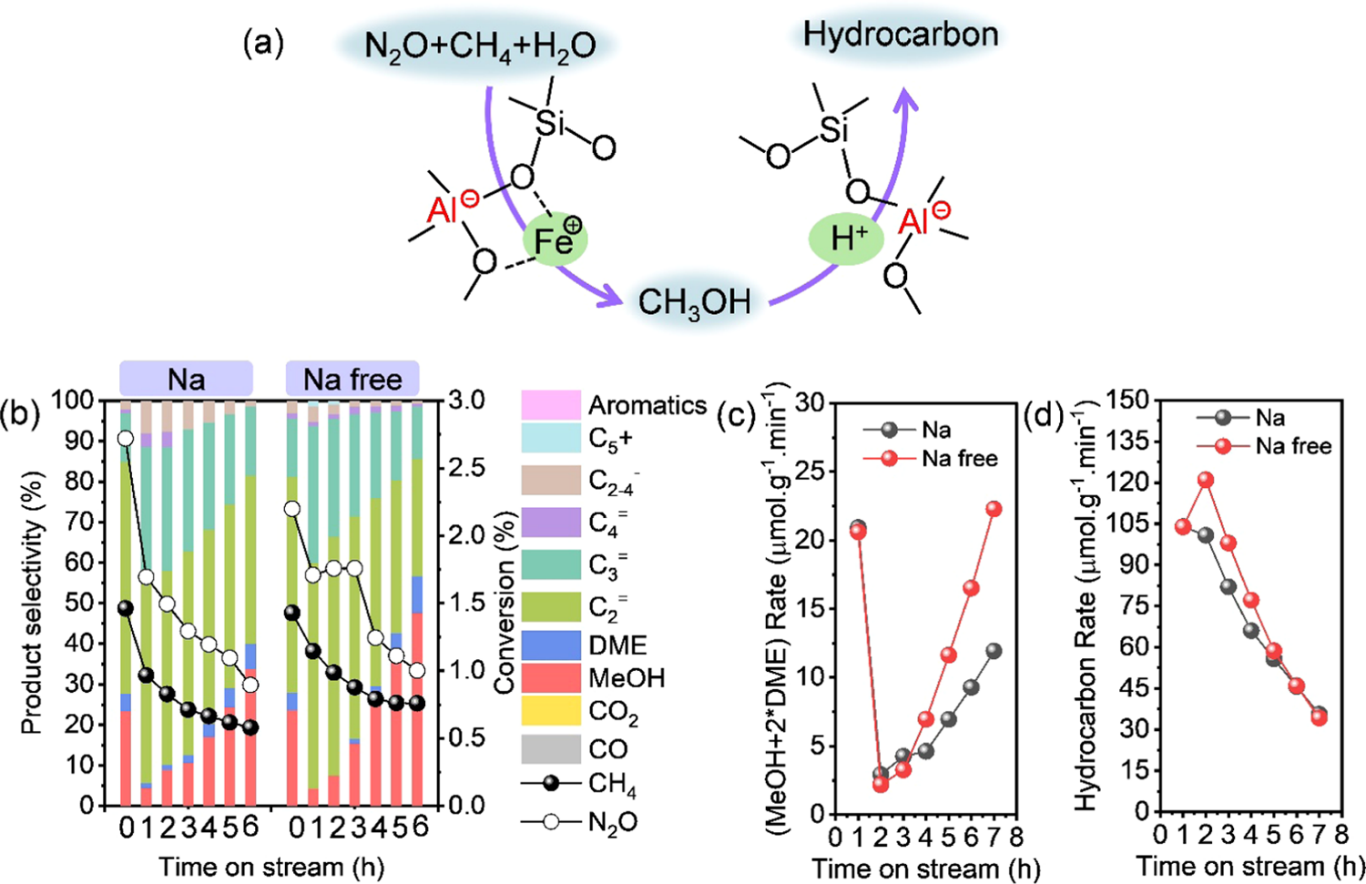
(a) Direct oxidation of methane to methanol on active Fe sites followed by the tandem conversion of methanol to hydrocarbon on acid sites over Fe-containing zeolites. Comparing (b) conversion and selectivity, (c) (MeOH + 2*DME) formation rate, and (d) hydrocarbon formation rate of H-type Fe-CHA(Na)-100 and Fe-CHA(Na free)-100 at 300 °C. Reaction conditions: 100 mg of catalyst, CH_4_/N_2_O/H_2_O/Ar = 10/10/2/3 mL·min^–1^, and WHSV = 15,000 mL·g^–1^·h^–1^.

As previously stated, 300 °C was the suitable temperature for the DMTM reaction on active Fe sites, immediately followed by the tandem conversion of MTH on acid sites over Fe-containing zeolites (Figure 6a).^38^ Therefore, the reaction was executed at 300 °C to investigate the activity of acid sites in the tandem oxidation of methane to hydrocarbon. As illustrated in Figure 6b, the maximum hydrocarbon selectivity of Fe-CHA(Na) and Fe-CHA(Na free) was up to 95%. Except for the initial methane conversion, Fe-CHA(Na free) achieved higher methane conversion and formation rate of (MeOH + 2*DME) and hydrocarbon than Fe-CHA(Na) (Figure 6c,d). Moreover, IE-Fe/CHA(Na free) and IE-Fe/CHA(Na) zeolites obtained 100% hydrocarbon selectivity under the same reaction conditions (Figure S20a). Similar to the one-pot-synthesized Fe-CHA zeolite, IE-Fe/CHA(Na free) also achieved higher methane conversion and higher formation rate of (MeOH + 2*DME) and hydrocarbon than IE-Fe/CHA(Na) (Figure S20b,c). Our previous work^11^ and the work from Corma and co-workers^10^ also disclosed that the isolated proton originating from Al_i_ gave rise to a longer lifetime in the MTH reaction. Thus, the isolated proton in the Fe-containing CHA(Na free) zeolite was concluded to exhibit a higher activity in the MTH reaction.

#### 3.2.3 Conversion of CH_4_ and N_2_O

**Figure 7:**
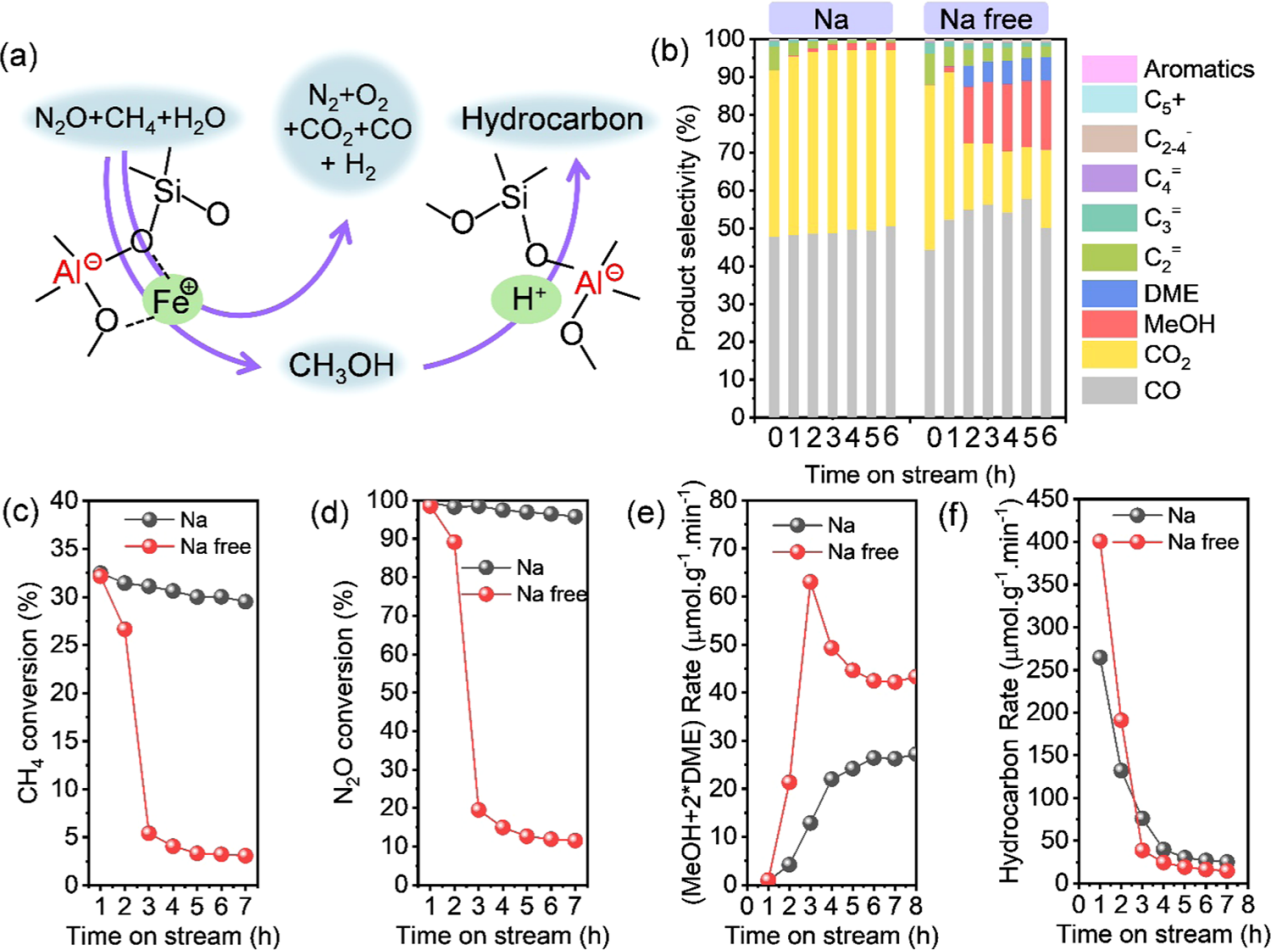
(a) Direct oxidation of methane to methanol and N_2_O decomposition on active Fe sites followed by the tandem conversion of methanol to hydrocarbon on acid sites over Fe-containing zeolites. Comparison of (b) product selectivity, (c) CH_4_ conversion, (d) N_2_O conversion, (e) (MeOH + 2*DME) formation rate, and (f) hydrocarbon formation rate of H-type Fe-CHA(Na)-100 and Fe-CHA(Na free)-100 at 350 °C. Reaction conditions: 100 mg of catalyst, CH_4_/N_2_O/H_2_O/Ar = 10/10/2/3 mL·min^–1^, and WHSV = 15,000 mL·g^–1^·h^–1^.

To comprehensively compare the activity of Fe-CHA zeolites with different Si/Fe ratios and Al arrangement, the oxidation of CH_4_ with N_2_O reaction was carried out at 350 °C. Under such high reaction temperatures, reactions including DMTM and N_2_O decomposition or the so-called N_2_O-SCR by CH_4_ on Fe sites^54^ and MTH reaction on acid sites occurred (Figure 7a). The high Si/Fe ratio of 800 led to the high hydrocarbon selectivity for both Fe-CHA(Na) and Fe-CHA(Na free) zeolites due to the insufficient Fe sites and sufficient acid sites (Figure S21a,b). The result was consistent with our previous work using Fe-AEI zeolites with different Si/Fe ratios.^38^ As the Si/Fe ratio decreased to 400, the hydrocarbon selectivity declined due to the increased Fe sites and, thus, the considerable methanol formation rate. Continuing to reduce the Si/Fe ratio to 100 and 50, the conversion of CH_4_ and N_2_O greatly increased to 33 and 100%, respectively, ignoring the Al arrangement (Figures 7 and S21). In general, higher conversion of CH_4_ and N_2_O was obtained over Fe-CHA(Na) zeolites than Fe-CHA(Na free) under a similar Fe content (Figure 7c,d). However, except for the individual cases, both the formation rate of (MeOH + 2*DME) and the hydrocarbon of Fe-CHA(Na free) were higher than those of Fe-CHA(Na) (Figures 7e,f, S21, and S22).

These results defended the higher activity of the isolated Fe site and isolated acidic site in the DMTM and MTH reactions, respectively, and the higher activity of proximal Fe sites in N_2_O decomposition. Fe-CHA(Na)-*x* obtained higher ethylene selectivity among hydrocarbons than that of Fe-CHA(Na free)-*x* (Figure S21g,h). The phenomenon can be explained by the side chain mechanism of the aromatic cycle due to the cooperative effects of the adjacent acid sites, which was consistent with the result of Plessow et al.^19^

Similarly, the IE-Fe/CHA zeolites displayed the same reaction performance at 350 °C (Figure S23). Specifically, IE-Fe/CHA(Na) achieved higher N_2_O conversion than IE-Fe/CHA(Na free) (Figure S23a). However, IE-Fe/CHA(Na free) obtained a higher formation rate of methanol and hydrocarbon than IE-Fe/CHA(Na) (Figures S23b,c and S24). Thus, we claimed that distant, isolated Fe and isolated protons in CHA(Na free) zeolites contributed to the higher activity in DMTM and MTH reactions than proximal, isolated Fe sites and paired protons in CHA(Na) zeolites. However, proximal Fe sites in IE-Fe/CHA(Na) zeolites evolving from Al_p_ contributed to higher N_2_O conversion in comparison to the isolated Fe in IE-Fe/CHA(Na free) zeolites. It has been reported that the proximal Fe sites were more active than the isolated Fe site in methane oxidation by comparing the N_2_O conversion.^39^ However, the N_2_O conversion in the presence of CH_4_ was related to CH_4_ consuming the oxygen of N_2_O to produce methanol and N_2_O decomposition. Therefore, it was not accurate to match the activity by comparing the N_2_O conversion in methane oxidation, especially at relatively high temperatures.

#### 3.2.4 N_2_O Decomposition

**Figure 8:**
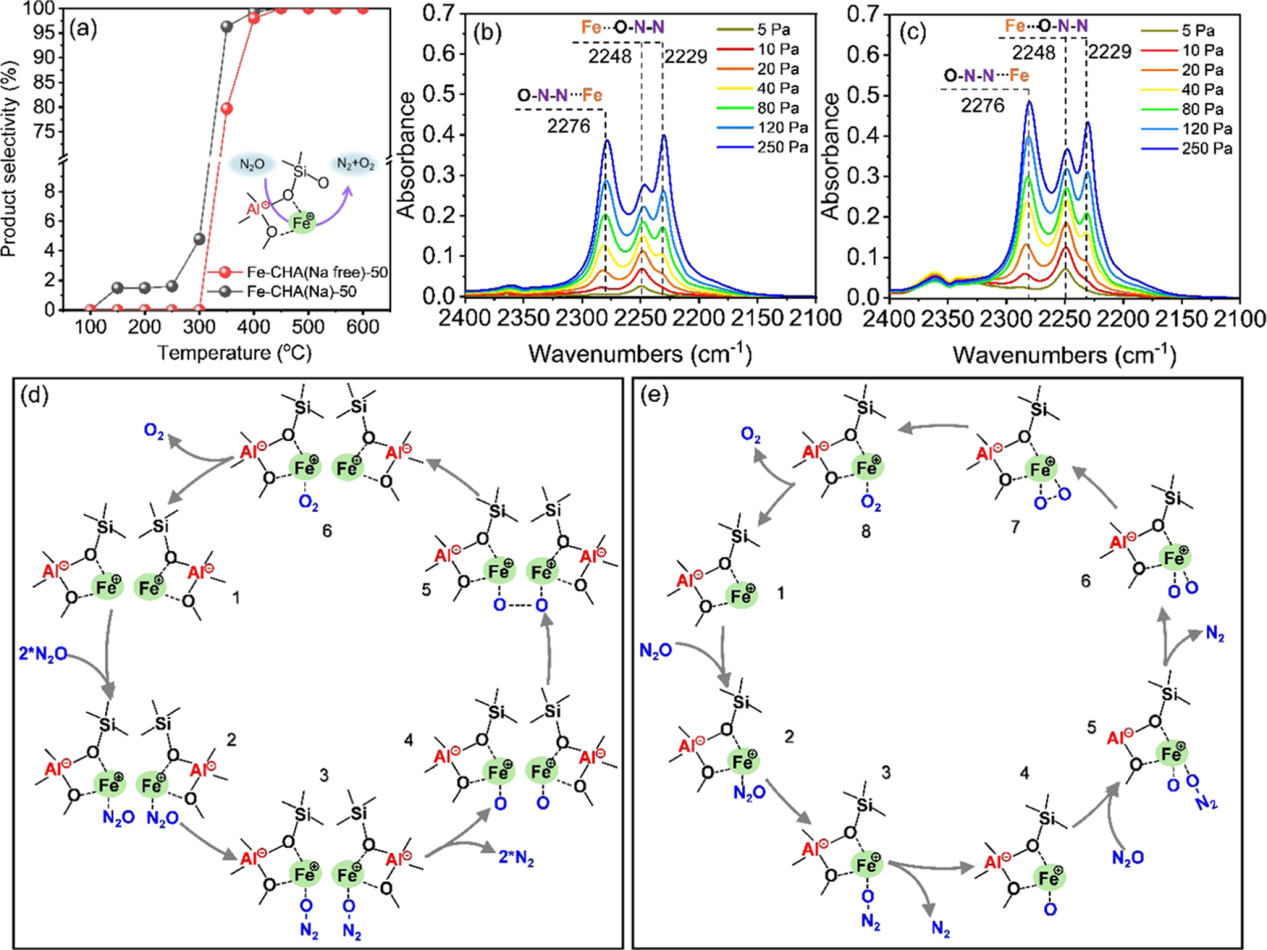
(a) Comparison of the N_2_O decomposition performance of Fe-CHA(Na)-50 and Fe-CHA(Na free)-50 at 100–600 °C. Reaction conditions: 100 mg of catalyst, N_2_O/Ar = 20/5 mL·min^–1^, and WHSV = 15,000 mL·g^–1^·h^–1^. N_2_O adsorption FTIR spectra (*P*_N_2_O_ = 5–250 Pa) at room temperature over (b) Fe-CHA(Na)-50 and (c) Fe-CHA(Na free)-50 zeolite catalysts after evacuation at 500 °C for 1 h. The possible pathway of N_2_O decomposition on (d) proximal Fe sites of Fe-CHA(Na) zeolite and (e) isolated Fe site of Fe-CHA(Na free) zeolite.

To shed light on the reason for the higher N_2_O conversion of Fe-containing CHA(Na) zeolites, N_2_O decomposition was conducted at 100–600 °C feeding only N_2_O and Ar using Fe-CHA(Na)-50 and Fe-CHA(Na free)-50 as catalysts representatively. As shown in Figure 8a, Fe-CHA (Na)-50 started to decompose N_2_O at 150 °C with a scant 1% conversion; subsequently, N_2_O conversion gradually increased and reached 100% at 400 °C. The N_2_O conversion of Fe-CHA(Na) at 150–400 °C was higher than that of Fe-CHA(Na free) due to the proximal Fe sites, being compatible with the literature.^55,56^ The N_2_O adsorption FTIR spectra were characterized on Fe-CHA(Na)-50 and Fe-CHA(Na free)-50 at 25 °C (Figure 8b,c). Fe-CHA(Na free)-50 presented a stronger intensity of the band at 2276 cm^–1^ than Fe-CHA(Na)-50, belonging to Fe-NNO components, which was not stable at high temperatures^57^ and not favorable for N_2_O decomposition.^58^ Moreover, the bands at 2229 and 2248 cm^–1^ were attributed to Fe-ONN and Al-ONN components, respectively (Figure S25), working as the active part of N_2_O decomposition.^59^

Additionally, Gao and co-workers exposed that Fe/CHA and Cu/CHA zeolites disclosed higher activity in N_2_O decomposition than the Co/CHA zeolite due to the proximal Fe or Cu sites in contrast to the isolated Co site.^59^ The information was valuable to interpreting the N_2_O decomposition performance on Fe-CHA(Na) and Fe-CHA(Na free) zeolites. Particularly, the proximal Fe sites (“1”) adsorbed two N_2_O (“2”) with the N–O cleavage on the active Fe sites (“3”) to form two “α-O” on the two vicinal Fe sites (“4”) after releasing two N_2_. Therefore, it was beneficial for the formation of O_2_ (“5”, “6”) (Figure 8d). Among these steps, N–O cleavage was recognized as the rate-limiting step. In contrast, for the isolated Fe site (1; Figure 8e), only one N_2_O was adsorbed on the isolated Fe at a time (“2”). The rate-limiting step of N–O cleavage occurred on the isolated Fe site (“3”) and only one “α-O” formed after the release of N_2_ (“4”). To generate O_2_, another rate-limiting step of N–O cleavage was required to occur on the same isolated Fe site (“5”). The previous steps (“1”–“4”) were continued (“5”, “6”) until two vicinal O atoms were connected (“7”). Finally, the formation and release of O_2_ were at an end (“8”). Based on the dual-site and single-site mechanisms, the N_2_O decomposition performance on the single Fe site was worse than on the proximal Fe sites restricted by two rate-limiting steps and the probability of N_2_O adsorbed on the same isolated Fe site.^59,60^ The discrepancy in N_2_O conversion can identify the isolated and proximal Fe sites. Therefore, the higher N_2_O conversion in the presence or absence of CH_4_ for Fe-containing CHA(Na) convincingly evidenced the proximal Fe sites.

### 3.3 Possible Pathway of Direct Oxidation of CH_4_ with N_2_O Affected by Al Arrangement

**Figure 9:**
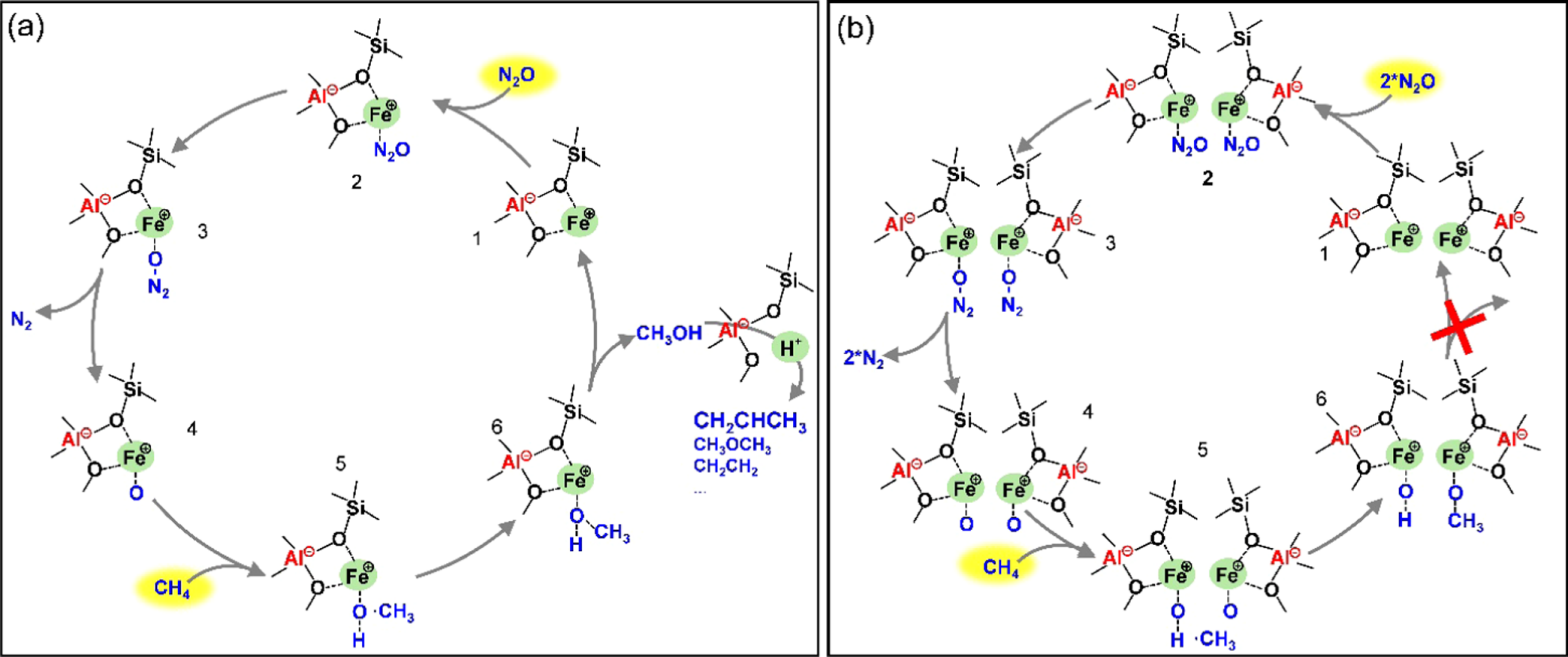
Possible pathway of direct oxidation of CH_4_ with N_2_O on (a) isolated Fe site and (b) proximal Fe sites without sufficient CH_4_.

About the reaction mechanism of direct oxidation of CH_4_, the radical rebound mechanism has been followed by researchers.^36,61−68^ Based on the literature^33−36^ and our recent work,^38^ DMTM reaction with N_2_O on Fe-containing zeolite experienced the following steps. Taking isolated Fe as an example (“1”, Figure 9a), first, N_2_O was adsorbed on the Fe site (“2”, Figure 9a); then, the bond of N–O was cleaved (“3”, Figure 9a), followed by releasing N_2_ and forming the active “α-O” site (“4”, Figure 9a). Afterward, CH_4_ was adsorbed on the active “α-O” site to produce a ^•^CH_3_ radical and an ^•^OH radical (“5”, Figure 9a); the methanol molecule was generated by the combination of ^•^CH_3_ and ^•^OH radicals (“6”, Figure 9a). When the acid sites were on the propagation path of methanol, methanol would be transformed to DME and hydrocarbon. However, in the case of proximal Fe sites, the reaction pathway depended on the reaction conditions. When the flow rate of CH_4_ and N_2_O was equivalent and only one of the proximal Fe sites took part in the reaction, the activity of proximal Fe sites should be comparable with that of the isolated Fe site (Figure S26). Nevertheless, paired protons caused a higher ethylene selectivity among hydrocarbons (Figure S26).^19^ When the flow rate of CH_4_ was less than that of N_2_O, the two formed proximate active “α-O” sites would compete for one ^•^CH_3_ and one ^•^OH radical. If the ^•^CH_3_ and ^•^OH radicals were adsorbed on the separated Fe sites, then, methanol was difficult to form, causing reduced activity (Figure 9b). To verify this hypothesis, the reaction performance of Fe-CHA(Na) and Fe-CHA(Na free) under the reaction conditions of CH_4_ and N_2_O flow rates of 5 and 15 mL·min^–1^, respectively, was compared (Figures S27 and S28). Both Fe-CHA(Na) and Fe-CHA(Na free) achieved 100% N_2_O conversion and 82% CH_4_ conversion (Figure S27a). However, the formation rate of (MeOH + 2*DME) and hydrocarbon for Fe-CHA(Na free) was higher than that of Fe-CHA(Na) (Figure S27b,c). These results verified the hypothesis. On the other hand, when the flow rate of CH_4_ was more than that of N_2_O, the formed two proximate active “α-O” sites would freely adsorb the sufficient ^•^CH_3_ and ^•^OH radicals, thus generating sufficient methanol and then continuing the tandem MTH reaction on the acid sites (Figure S29). The higher conversion including CH_4_ and N_2_O and higher formation rate including (MeOH + 2*DME) and hydrocarbons of Fe-CHA(Na) than those of Fe-CHA(Na free) with the CH_4_ and N_2_O flow rates of 15 and 7.5 mL·min^–1^, respectively, strongly supported the hypothesis mentioned above when the reaction stabilized (Figures S30 and S31).

## 4 Conclusions

Fe-containing aluminosilicate CHA zeolites with Fe species at different spatial distances affected by the Al arrangement were prepared in the presence or absence of Na. The Al arrangement was identified by ^27^Al and ^29^Si MAS NMR spectra and TG-DTA curves. UV–vis, XAS, and NO adsorption FTIR spectra were used to analyze Fe speciation. NO adsorption FTIR spectra showed that the higher proportion of Fe species for Fe-CHA(Na) was in 6 MR. The higher N_2_O conversion further confirmed the proximal Fe sites of Fe-CHA(Na). The activities of different spatial distance Fe sites in reactions, including direct oxidation of methane to methanol, methanol to hydrocarbon, and N_2_O decomposition, were compared to provide the active sites and reaction pathways. The isolated Fe and isolated protons were more active in DMTM and tandem conversion of MTH reactions than the proximal Fe sites and paired protons, respectively. The proximal Fe sites exhibited higher activity in N_2_O decomposition than the isolated Fe. This study revealed the active sites and reaction pathways in the direct oxidation of methane over Fe-containing zeolites. Our findings guided the preparation of active catalysts for oxidation of methane to methanol and methanol to hydrocarbon and N_2_O decomposition, significantly addressing energy and environmental concerns.

## Supporting Information Available

Experimental details; methods, characterization results of solid ^29^Si and ^27^Al MAS NMR; N_2_ adsorption and desorption; NH_3_-TPD spectra; CO adsorption FTIR spectra; HAADF-STEM images; corresponding EDS elemental mapping images; HRTEM image; iDPC-STEM images; information on one-pot-synthesized Fe-CHA zeolites; Fe-exchanged CHA zeolites, and reaction performance of other catalysts (PDF)
