# Supplementary material for: Revealing Active Sites and Reaction Pathways in Direct Oxidation of Methane over Fe-Containing CHA Zeolites Affected by the Al Arrangement
Source: J Am Chem Soc. 2024 Nov 5;146(46):31969–81. doi: 10.1021/jacs.4c11773 (PMC11583304; doi:10.1021/jacs.4c11773)
Supplement: Supplementary file 1 — ja4c11773_si_001.pdf [file ja4c11773_si_001.pdf]

Supporting information

Revealing active sites and reaction pathways in direct oxidation of methane over Fe-containing CHA zeolites affected by Al arrangement

Peipei Xiao [a], Lizhuo Wang [b], Hiroto Toyoda [a], Yong Wang [a], Kengo Nakamura [a], Jun Huang [b], Ryota Osuga [c], Maiko Nishibori [d], Hermann Gies [a], Toshiyuki Yokoi [a,e]*

[a] Institute of Innovative Research, Tokyo Institute of Technology, 4259 Nagatsuta, Midori-ku, Yokohama 226-8501, Japan

[b] School of Chemical and Biomolecular Engineering, the University of Sydney, Sydney, New South Wales 2006, Australia

[c] Institute for Catalysis, Hokkaido University, Kita 21 Nishi 10, Kita-ku, Sapporo, Hokkaido 001-0021, Japan

[d] International Center for Synchrotron Radiation Innovation Smart, Tohoku University, 2-1-1 Katahira, Aoba-ku, Sendai, Miyagi, 980-8577, Japan

[e] iPEACE223 Inc., Konwa Building, 1-12-22 Tsukiji, Chuo-ku, Tokyo, 104-0045, Japan

*E-mail: yokoi@cat.res.titech.ac.jp

Supplementary Methods

Catalyst characterization

XRD pattern was collected on a Rint-Ultima III (Rigaku) using a Cu Kα X-ray source (40 kV, 40 mA).

Elemental analyses of the samples were performed on an inductively coupled plasma-atomic emission spectrometer (ICP-AES, Shimadzu ICPE-9000).

Field-emission scanning electron microscopic (FE-SEM) images of the powder samples were obtained on the SU-9000 (Hitachi) microscope operating at 1 kV.

High-angle annular dark field scanning transmission electron microscopy (HAADF-STEM) imaging, integrated differential phase contrast STEM (STEM-iDPC) imaging, and energy dispersive spectrometry (EDS) mapping were performed on a FEI Themis Z microscope equipped with an XFEG field electron source and double aberration corrector operated on 300 keV. The HAADF-STEM images were acquired with a camera length of 115 mm while the beam convergence was 25.1 mrads. The pixel size is 37 pm and the dwell time is 2 us/pixel. The collection angle of the HAADF detector was set to 48–200 mrads. STEM-EDS elemental maps were acquired with 4 us/pixel acquisition time using Velox software. High-resolution transmission electron microscopy (HRTEM) images were acquired on the same image operated on 300 keV.

Nitrogen adsorption and desorption measurements to obtain the information on the micro- and meso-porosities were conducted at -196 ^{o}C on a Belsorp-mini II (MicrotracBEL).

The amount of OSDA in the as-synthesized samples was determined by the weight loss from 250 to 800 ^{o}C in a thermogravimetric (TG) profile, which was performed on a thermogravimetric-differential thermal analyzer (TG-DTA, RigakuThermo plus EVO II).

Solid-state ^{29}Si MAS NMR, ^{29} Si CPMAS NMR, and ^{27}Al MAS NMR spectra were measured on a JEOL ECA-600 spectrometer at a resonance frequency of 156.4 MHz using a 4 mm sample rotor with a spinning rate of 15.0 kHz. The ^{29}Si and ^{27}Al chemical shifts were referenced to -34.12 and -0.54 ppm, polydimethylsiloxane (PDMS) and $AlNH_4(SO_4)_2 \cdot 12H_2O$, respectively.

Temperature-programmed ammonia desorption (NH_3-TPD) profiles were recorded on Multitrack TPD equipment (Japan BEL). Typically, 25 mg of catalyst was pretreated at 600 ^{o}C in He flow (50 mL min^{-1}) for 1 h and then cooled to 100 ^{o}C. Prior to the adsorption of NH_3, the sample was evacuated at 100 ^{o}C for 1 h. Approximately 2500 Pa of NH_3 was allowed to contact with the sample at 100 ^{o}C for 10 min. Subsequently, the sample was evacuated to remove weakly adsorbed NH_3 at the same temperature for 30 min. Finally, the sample was cooled to 100 ^{o}C and heated from 100 to 600 ^{o}C at a ramping rate of 10 ^{o}C min^{-1} in a He flow (50 mL min^{-1}). A thermal conductivity detector (TCD) was employed to monitor desorbed NH_3. The amount of acid sites was determined by the fitting peak area.

Fe K-edge X-ray absorption spectroscopy (XAS) was conducted with a transmission mode to characterize the oxidation state and the local structure of Fe species. Spectra were obtained using synchrotron radiation at the SPring-8 BL01B1 beamline in Hyogo, Japan, equipped with a Si(111) double-crystal monochromator, 100% N_2 for I_0 ion chamber and 15% Ar/N_2 for I_1 ion chamber. The obtained XAS spectra were analyzed using Athena software version 0.9.26.[1] The oscillations of k^2-weighted Fe K-edge extended X-ray absorption fine structure (EXAFS) were Fourier-transformed (FT) in the k range from 3.0 to 10.0 Å^{-1}.[1]

UV-vis spectra were collected in the range of 190-800 nm on a V-650DS spectrometer (JASCO). The diffuse reflectance spectra were converted into the absorption spectra using the Kubelka-Munk function.

The Fe species was analyzed via FTIR analysis using NO and CO as the probe molecules. The samples were pressed into a self-supporting disk (20 mm diameter, 30−60 mg) and placed in an IR cell

attached to a closed-gas circulation system. TGS detector was used. The samples were degassed under the vacuum condition at 500 °C for 1 h to remove adsorbed water. The adsorption of NO with different pressures (5-80 Pa) and CO with different pressures (5-1000 Pa) was performed at -120 ^{o}C.

The N_2O adsorption FTIR was carried out in the FTIR system by using a JASCO 4100 FTIR spectrometer equipped with a triglycine sulfate (TGS) detector. IR spectra of the clean disk were recorded in vacuo at 25 ^{o}C to obtain background spectrum. The sample was pressed into a self-supporting disk (20 mm diameter, 30−60 mg) and placed in an IR cell attached to a closed-gas circulation system. The sample was pretreated by evacuation at 500 ^{o}C for 1 h, followed by adsorption of 5−250 Pa N_2O at 25 ^{o}C.

Catalytic activity test

The continuous oxidation of methane reaction was performed in a fixed-bed flow reactor (Figure S6). Typically, 100 mg of catalyst in a pellet form (pellet size 500−1000 μm) was charged into a quartz tube (inner diameter 4 mm), which was placed in an electric tube furnace. The catalyst was pretreated at 500 °C for 1 h. The reaction was conducted at typically 350 °C (Figure S7). The outlet gas, containing the products, unreacted CH_4 and N_2O were analyzed using two on-line gas chromatographs (GC; GC-2014, Shimadzu). One of the GCs was equipped with a Shincarbon ST 50/80 packed column (3 mm×6 m) and a TCD detector. Specifically, GC-TCD with a methanizer was employed to detect H_2, N_2O, CO, CO_2, and CH_4. The other GC was equipped with a HP-PLOT Q packed column (0.53 mm×30 m×40 μm), a flame ionization detector (FID). The GC-FID was used to investigate CH_4, and the produced methanol (MeOH), dimethyl ether (DME), low olefins and alkanes. The yield of each carbon-containing product was calculated by considering the number of carbon atoms. The methane conversion in this study was defined as the total obtained products, and calculated as:

$$C_{\mathrm{CH4}} = \frac{\Sigma(i*Ci)}{\Sigma(i*Ci)+CH4}$$

where C_{CH4} is the CH_4 conversion, i is the number of carbon atoms in product C_i, meanwhile, C_i *is the* carbon amount of product C_i, $\Sigma(i*C_i)$ is the total carbon amount of all the carbon-containing products, and $CH4$ is the amount of CH_4 detected at the same time.

The N_2O conversion was calculated as:

$$C_{\mathrm{N2O}} = \frac{ni-na}{ni}$$

where C_{N2O} is the N_2O conversion, n_i is the initial N_2O molar weight, n_a is the N_2O molar weight after reaction.

The product selectivity is calculated as:

$$S_{\mathrm{Ci}} = \frac{i*Ci}{\Sigma(i*Ci)}$$

where S_{Ci} is the selectivity of the product C_i, i is the number of carbon atoms in product C_i, $\Sigma(i*C_i)$ is the total carbon amount of all the products.

The product yield is calculated as:

$$Y_{\mathrm{Ci}} = \frac{i*Ci}{\Sigma(i*Ci)+CH4}$$

where Y_{Ci} is the yield of the product C_i, i is the number of carbon atoms in product C_i, $\Sigma(i*C_i)$ is the total carbon amount of all the products, and $CH4$ is the amount of CH_4 detected at the same time.

The product formation rates are calculated as:

$$R_{\mathrm{Ci}} = Y_{\mathrm{Ci}} * F_{\mathrm{CH4}}/m_{cat}$$

where R_{Ci} is the formation rate of product C_i, Y_{Ci} is the yield of the product C_i, F_{CH4} is the initial flow rate of CH_4, m_{cat} is the mass of the catalyst.

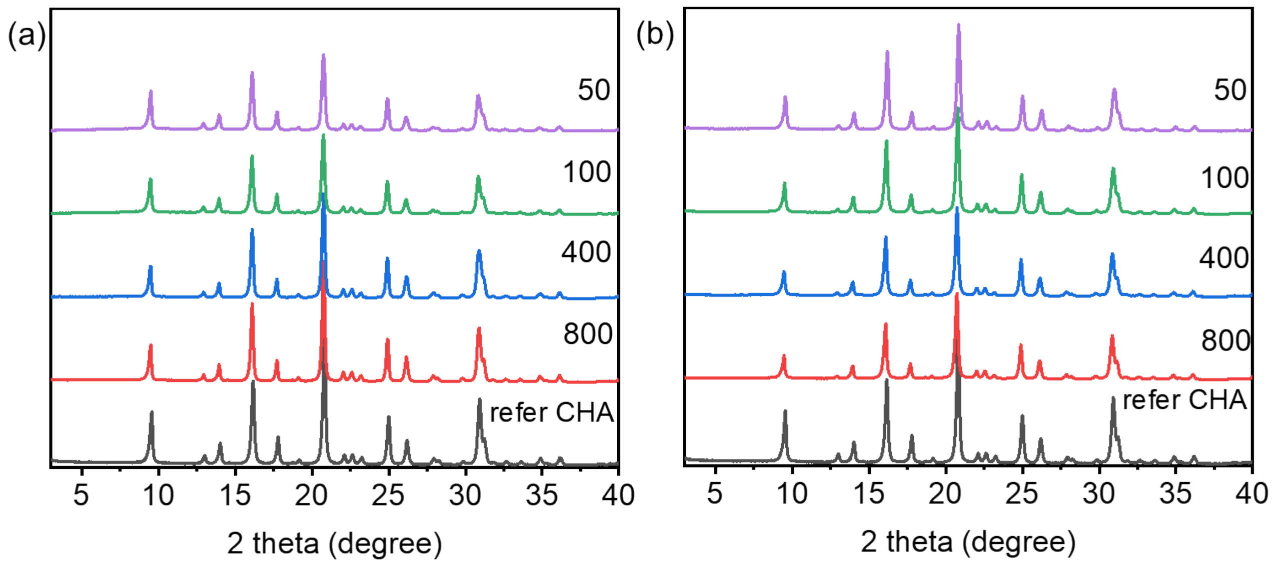

Figure S1. XRD patterns of as-synthesized (a) Fe-CHA(Na)-*x* and (b) Fe-CHA(Na free)-*x*, where *x* means the Si/Fe ratio in the synthesis gel of 800, 400, 100, and 50.

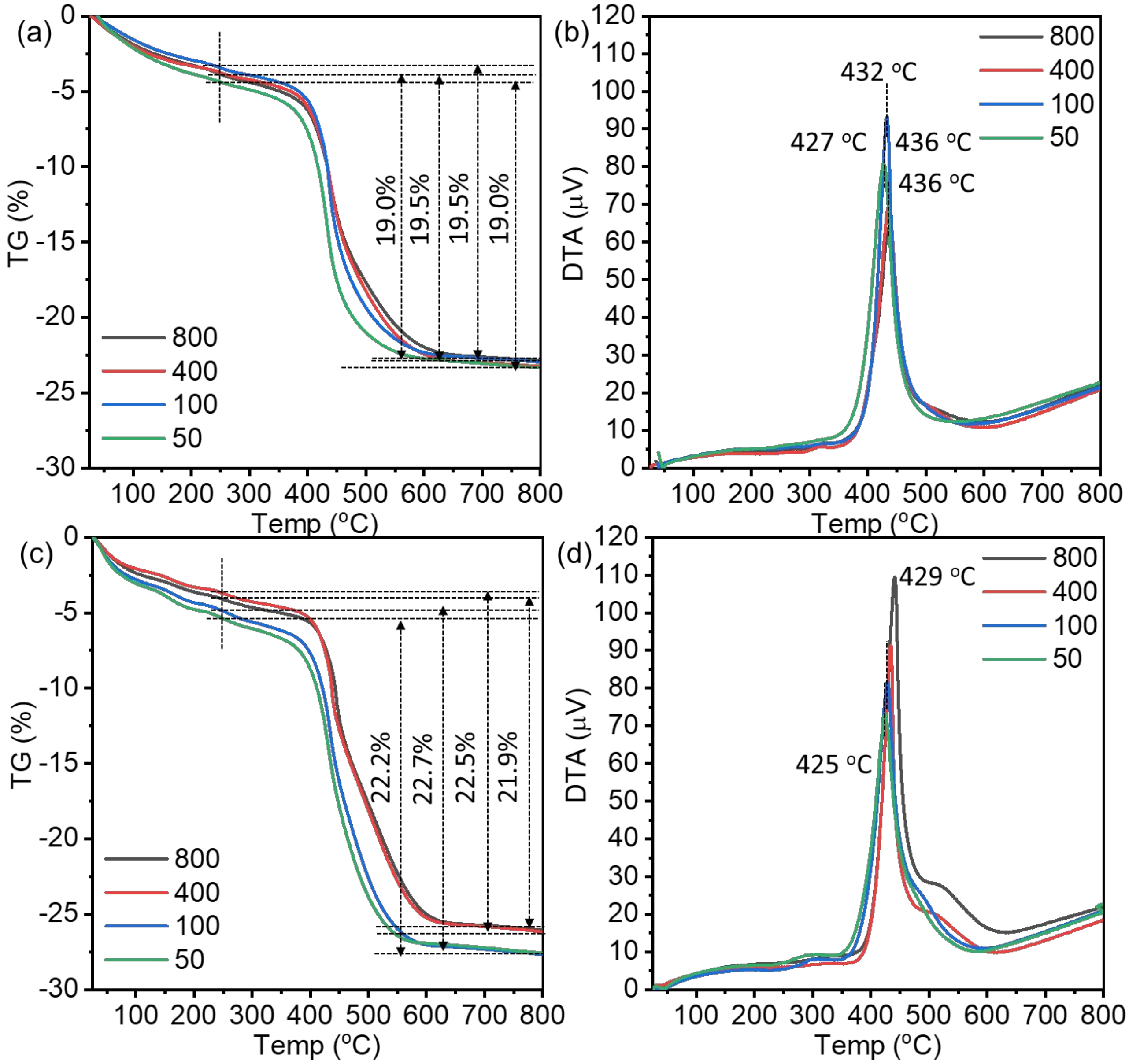

Figure S2. (a) TG and (b) DTA curves of the as-synthesized Fe-CHA(Na)-*x* zeolites. (c) TG and (d) DTA curves of the as-synthesized Fe-CHA(Na free)-*x* zeolites, where *x* means the Si/Fe ratio in the synthesis gel of 800, 400, 100, and 50.

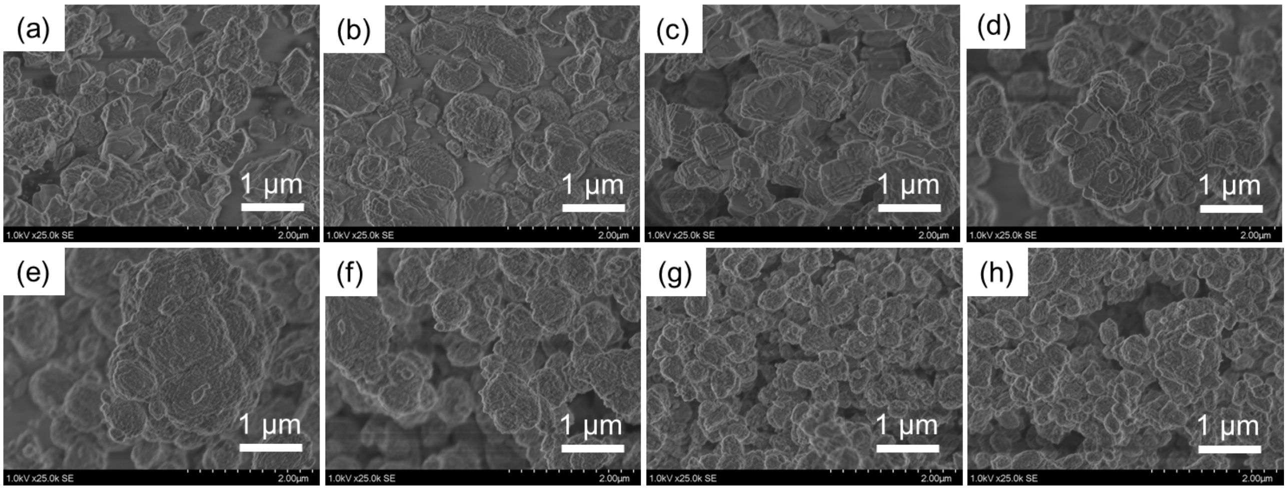

Figure S3. SEM images of (a) Fe-CHA(Na)-800, (b) Fe-CHA(Na)-400, (c) Fe-CHA(Na)-100, (d) Fe-CHA(Na)-50, (e) Fe-CHA(Na free)-800, (f) Fe-CHA(Na free)-400, (g) Fe-CHA(Na free)-100, and (h) Fe-CHA(Na free)-50.

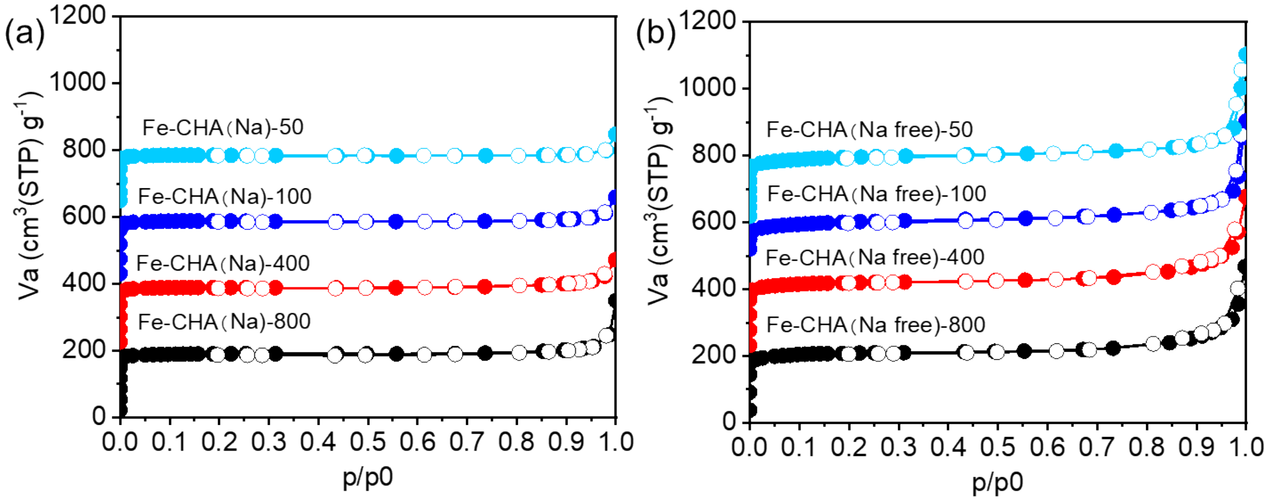

Figure S4. N_2 adsorption and desorption isotherms of H-type (a) Fe-CHA(Na)-*x* and (b) Fe-CHA(Na free)-*x* zeolites, where *x* means the Si/Fe ratio in the synthesis gel of 800, 400, 100, and 50. The isotherms for Fe-CHA-400, 100, and 50 were offset vertically by 200, 400, and 600 $cm^3 \cdot g^{-1}$, respectively.

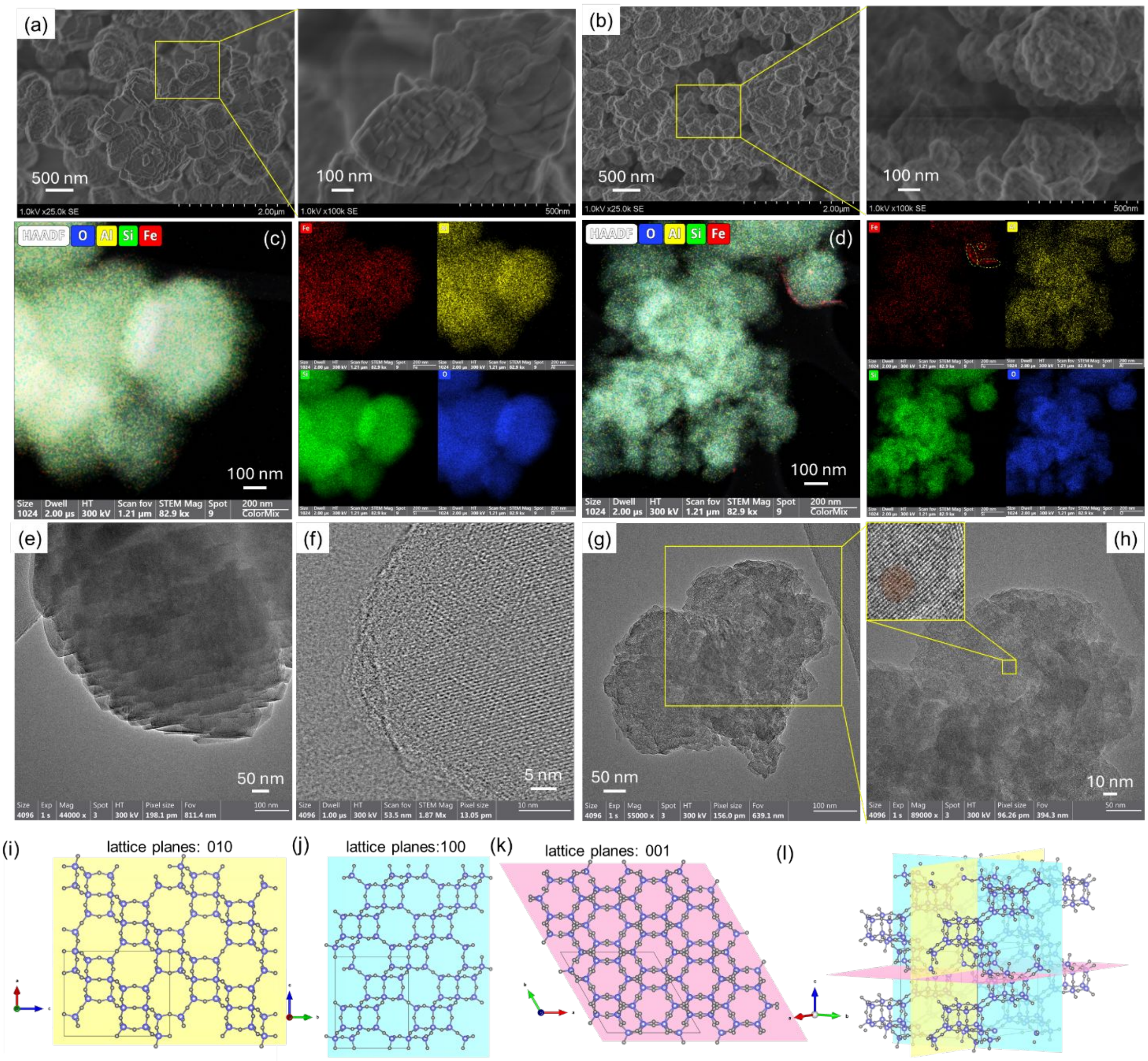

Figure S5. SEM images of (a) Fe-CHA(Na)-50 and (b) Fe-CHA(Na free)-50. HAADF-STEM images and corresponding EDS elements mapping images of (c) Fe-CHA(Na)-50 and (d) Fe-CHA(Na free)-50. HRTEM images of (e) Fe-CHA(Na)-50 and (g) Fe-CHA(Na free)-50. iDPC-STEM images of (f) Fe-CHA(Na)-50 and (h) Fe-CHA(Na free)-50. Lattice planes of CHA zeolite projection along (i) 010, (j) 100, (k) 001, and (l) the three lattice planes are shown together.

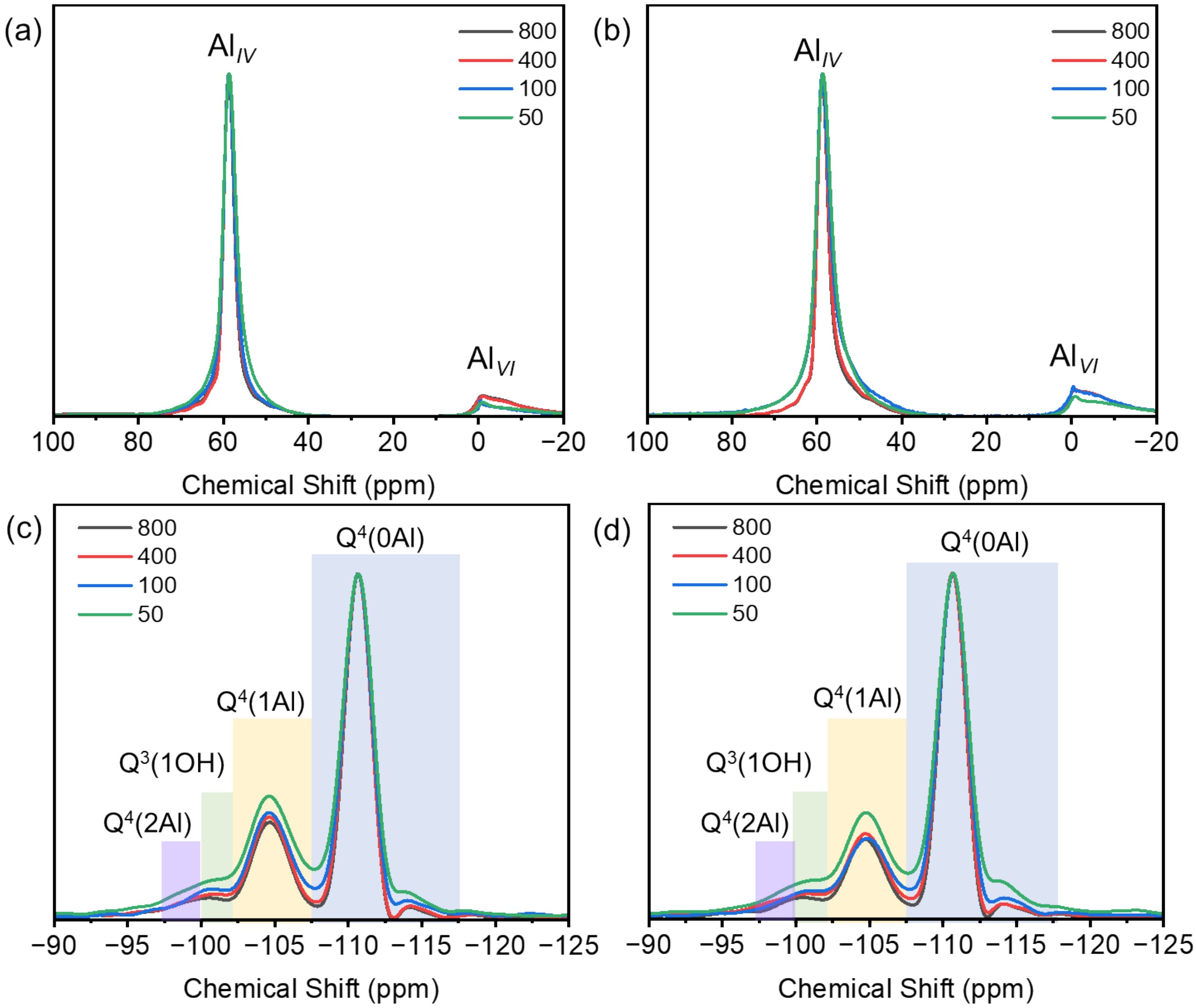

Figure S6. ^{27}Al MAS NMR spectra for H-type (a) Fe-CHA(Na)-*x*, and (b) Fe-CHA(Na free)-*x*. ^{29}Si MAS NMR spectra for H-type (c) Fe-CHA(Na)-*x*, and (d) Fe-CHA(Na free)-*x*, where *x* means the Si/Fe ratio in the synthesis gel of 800, 400, 100, and 50.

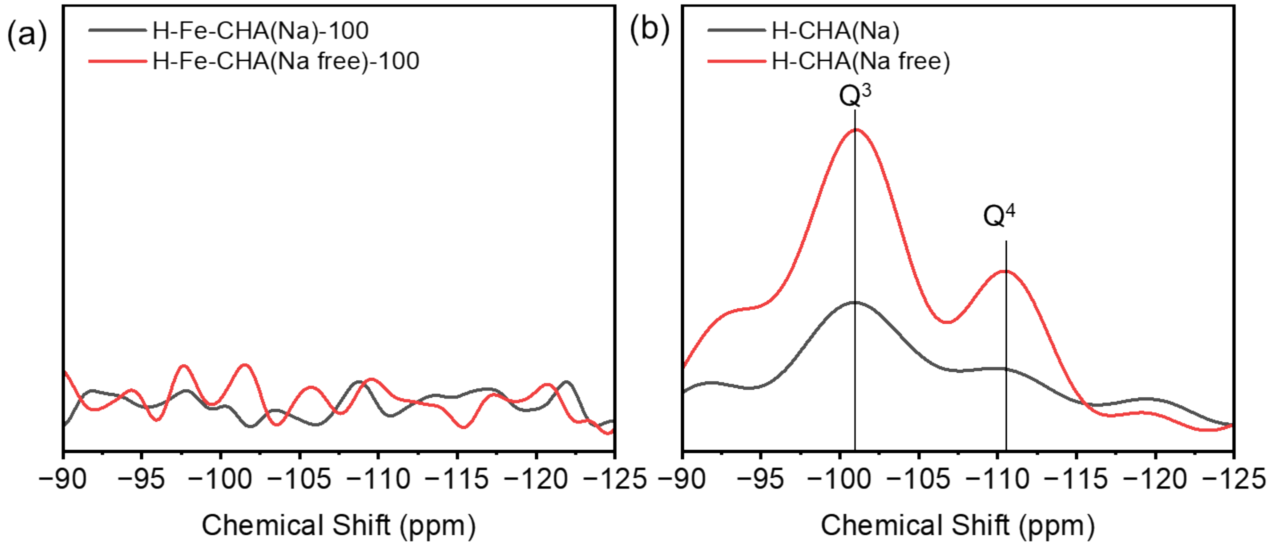

Figure S7. ^{29}Si CPMAS NMR spectra for H-type (a) Fe-CHA(Na)-100 and Fe-CHA(Na free)-100 and (b) CHA(Na) and CHA(Na free).

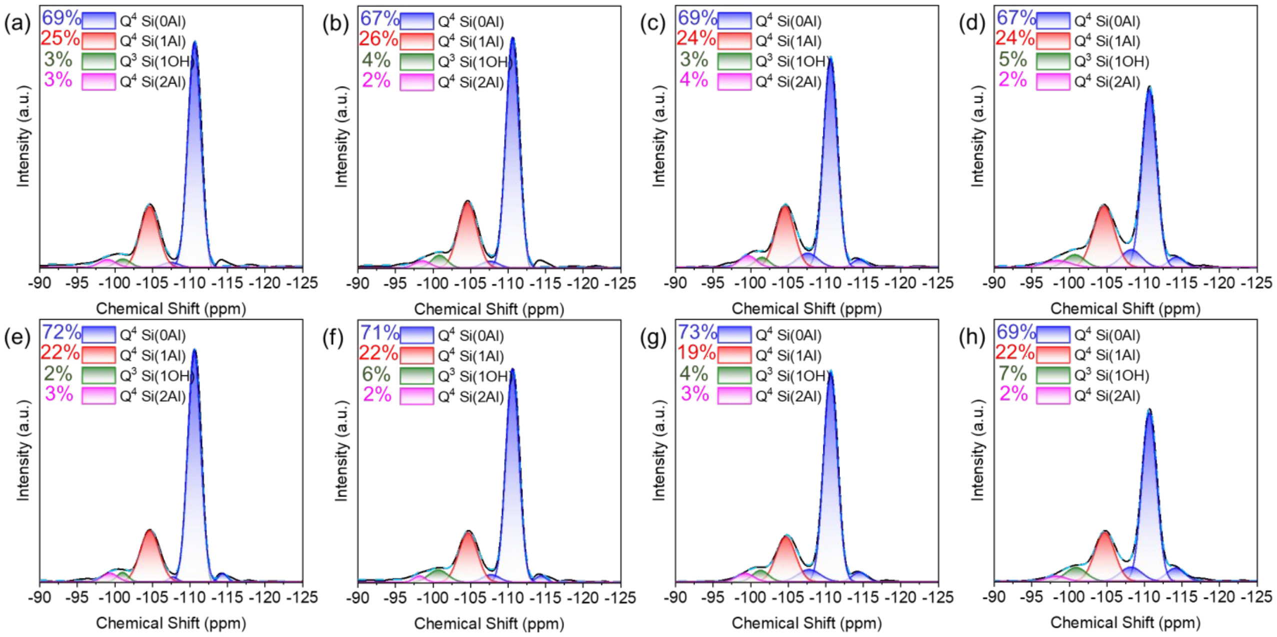

Figure S8. Deconvolution of ^{29}Si MAS NMR spectra for H-type (a) Fe-CHA(Na)-800, (b) Fe-CHA(Na)-400, (c) Fe-CHA(Na)-100, (d) Fe-CHA(Na)-50, (e) Fe-CHA(Na free)-800, (f) Fe-CHA(Na free)-400, (g) Fe-CHA(Na free)-100, and (h) Fe-CHA(Na free)-50.

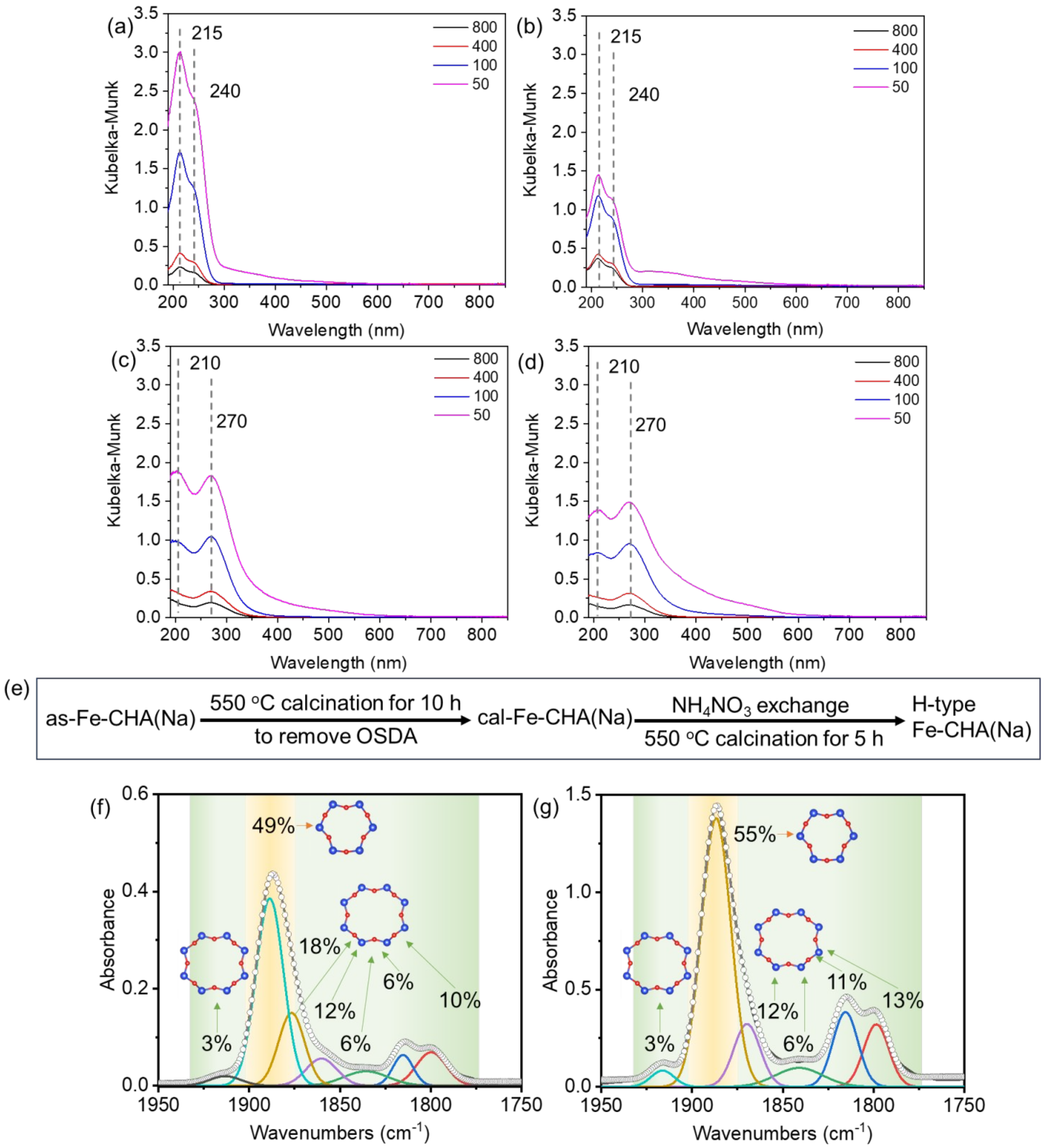

Figure S9. UV-vis spectra of as-synthesized (a) Fe-CHA(Na)-*x* and (b) Fe-CHA(Na free)-*x*, H-type (c) Fe-CHA(Na)-*x* and (d) Fe-CHA(Na free)-*x*, where *x* means the Si/Fe ratio in the synthesis gel of 800, 400, 100, and 50. (e) Preparation process from as-Fe-CHA(Na)-100 to cal-Fe-CHA(Na)-100 followed by H-type Fe-CHA(Na). Deconvolution of NO adsorption (P_{NO} = 80 Pa) FTIR spectra for (f) cal-Fe-CHA(Na)-100 and (g) H-type Fe-CHA(Na)-100.

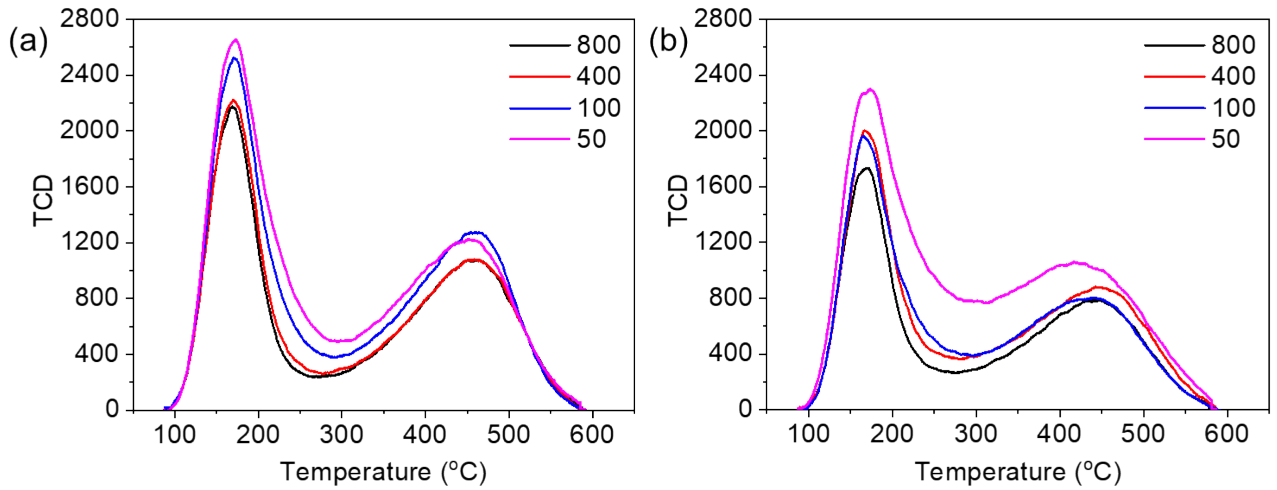

Figure S10. NH_3-TPD curves of the H-type (a) Fe-CHA(Na)-*x* and (b) Fe-CHA(Na free)-*x* zeolite catalysts, where *x* means the Si/Fe ratio in the synthesis gel of 800, 400, 100, and 50.

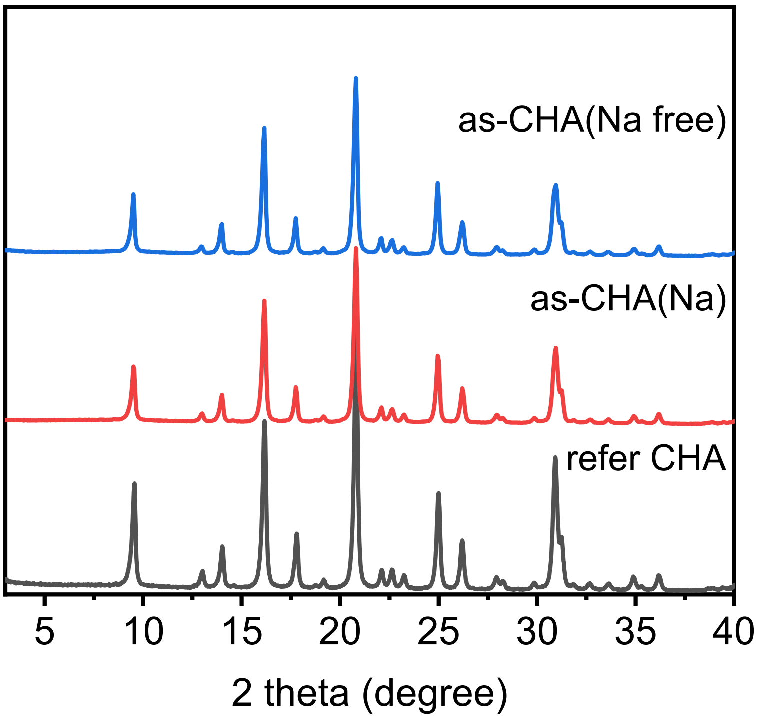

Figure S11. XRD patterns of as-synthesized CHA(Na) and CHA(Na free) zeolites.

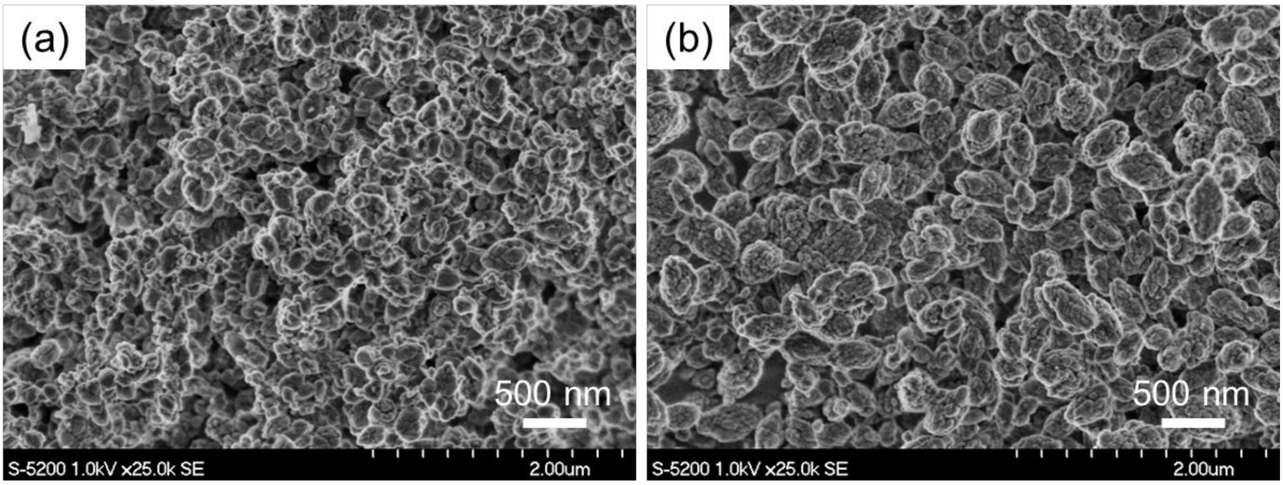

Figure S12. SEM images of (a) CHA(Na) and (b) CHA(Na free).

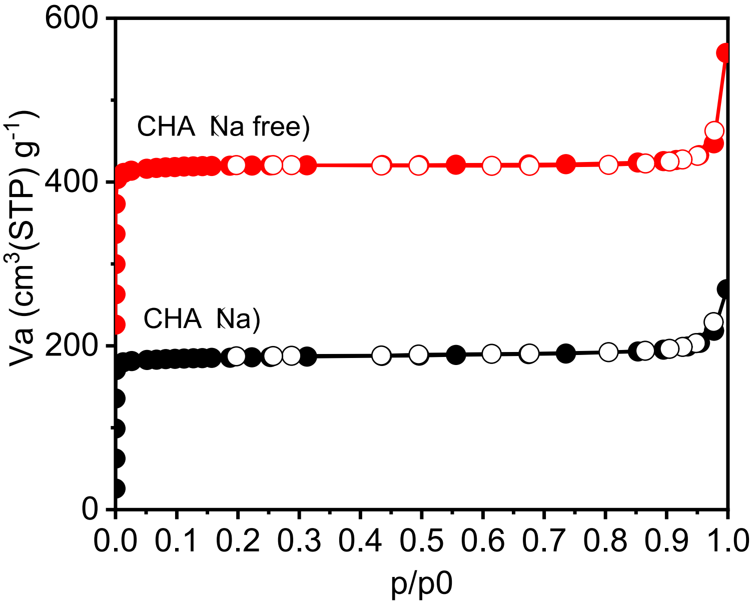

Figure S13. N_2 adsorption and desorption isotherms of H-type IE-Fe/CHA(Na) and IE-Fe/CHA(Na free) zeolites. The isotherm for IE-Fe/CHA(Na free) was offset vertically by 200 $cm^3 \cdot g^{-1}$.

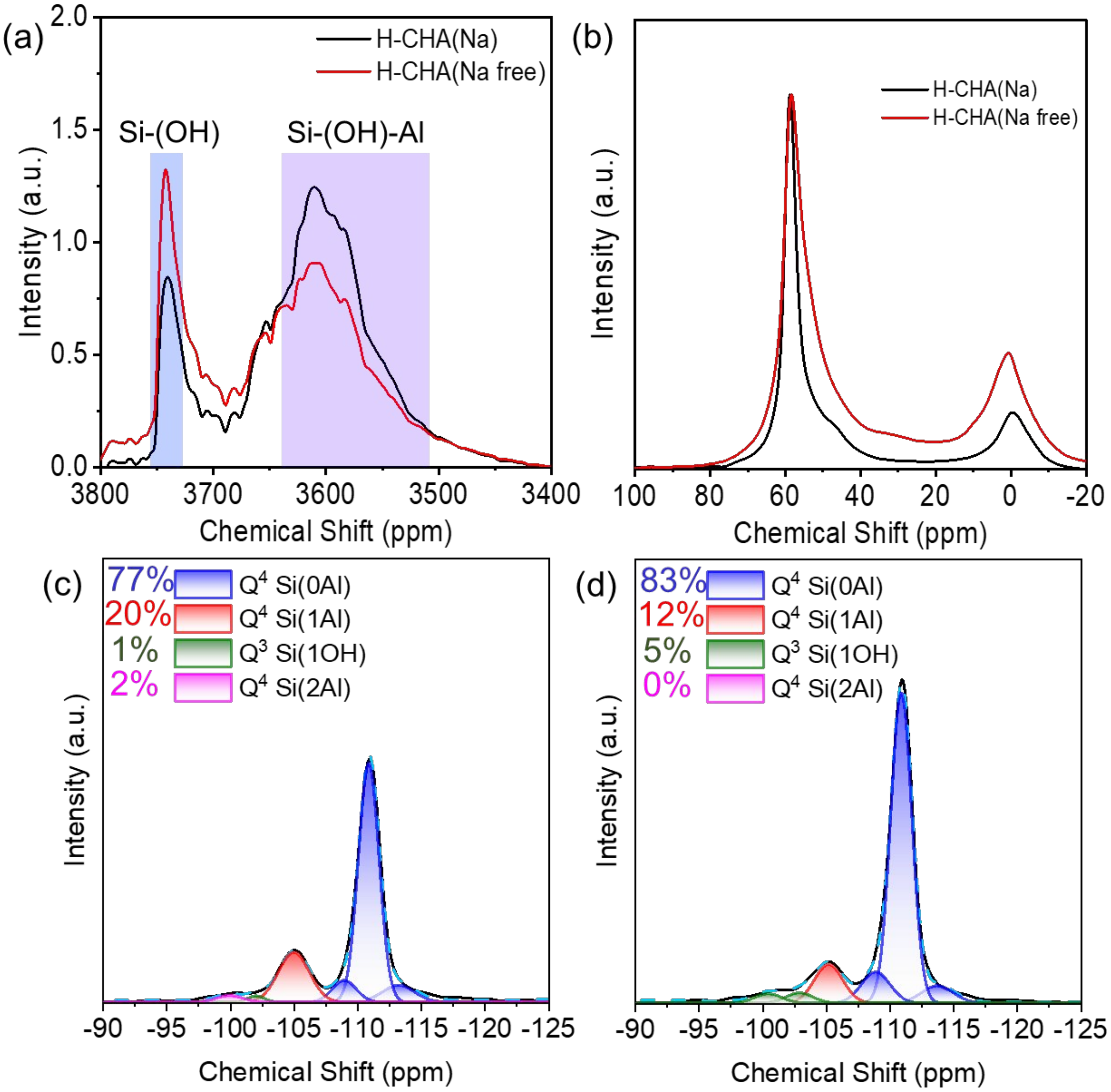

Figure S14. (a) Hydroxyl vibration at room temperature after evacuation at 500 ℃ for 1 h, (b) ^{27}Al MAS NMR spectra for H-type CHA(Na) and CHA(Na free). Deconvolution of ^{29} Si MAS NMR spectra for H-type (c) CHA(Na) and (d) CHA(Na free).

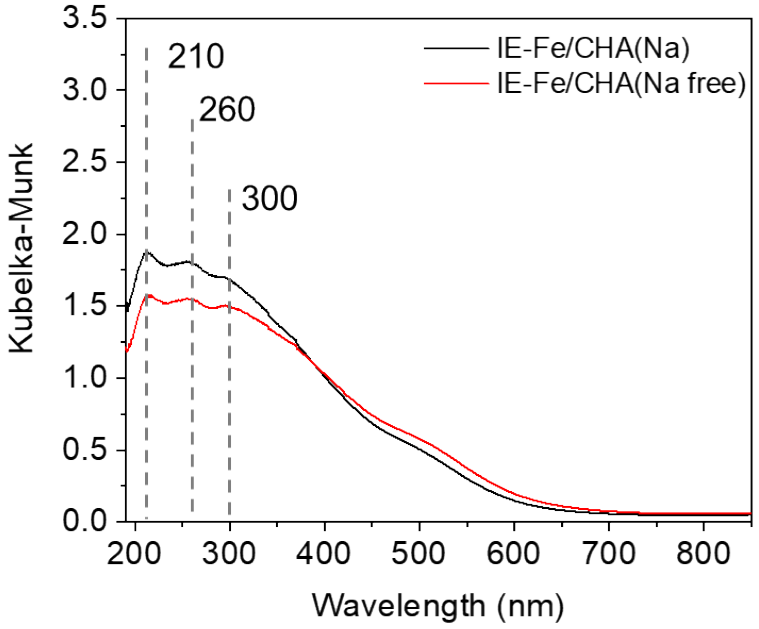

Figure S15. UV-vis spectra for IE-Fe/CHA(Na) and IE-Fe/CHA(Na free).

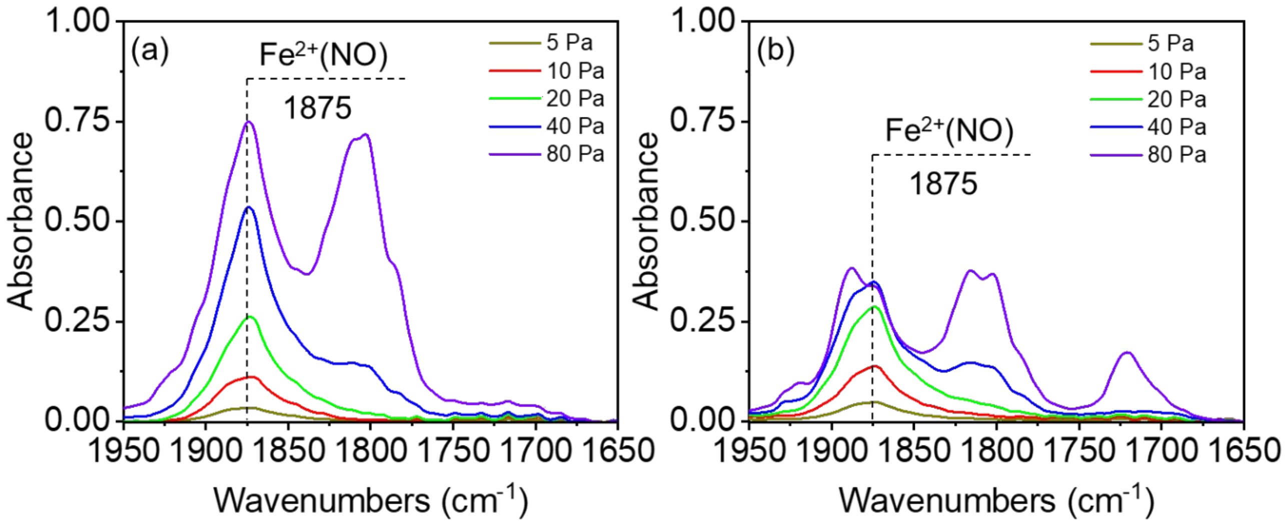

Figure S16. NO adsorption FTIR spectra (P_{CO}=5-80 Pa) at -120 ℃ after evacuation at 500 ℃ for 1 h for (a) IE-Fe/CHA(Na) and (b) IE-Fe/CHA(Na free) zeolite catalysts.

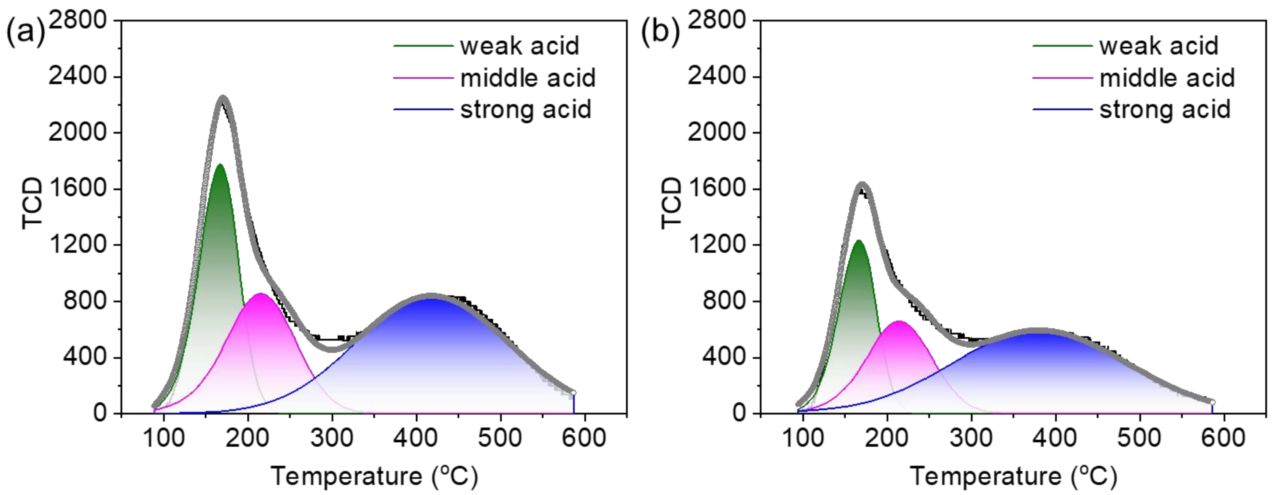

Figure S17. Deconvolution of NH_3-TPD curves for (a) IE-Fe/CHA(Na) and (b) IE-Fe/CHA(Na free).

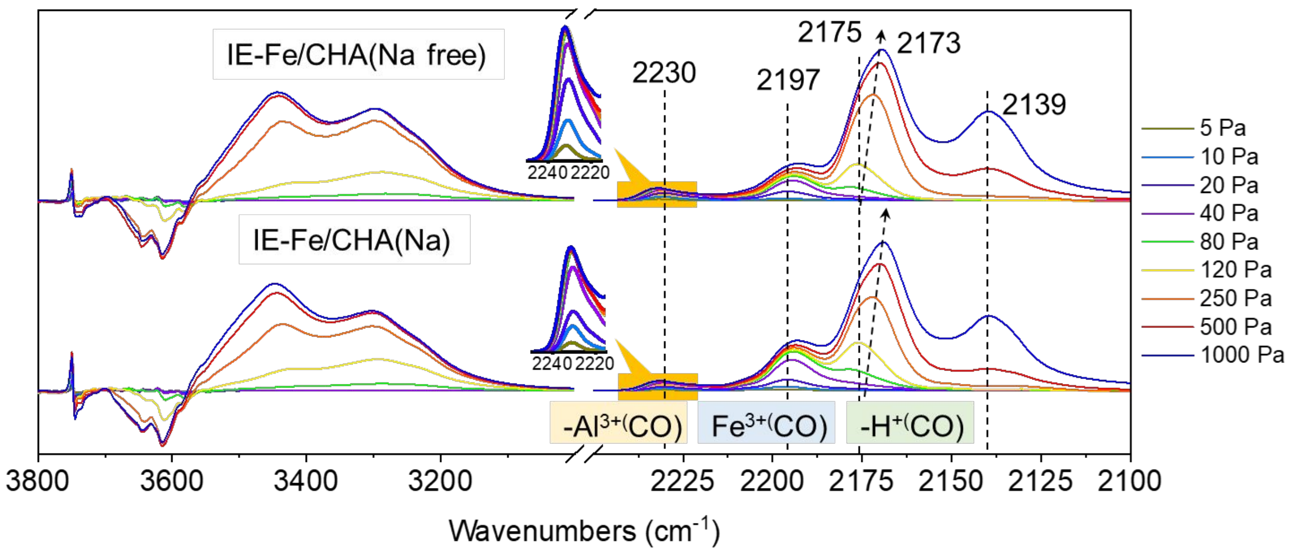

Figure S18. CO adsorption FTIR spectra (P_{CO}=5-1000 Pa) at -120 ℃ after evacuation at 500 ℃ for 1 h for IE-Fe/CHA(Na) and IE-Fe/CHA(Na free) zeolite catalysts.

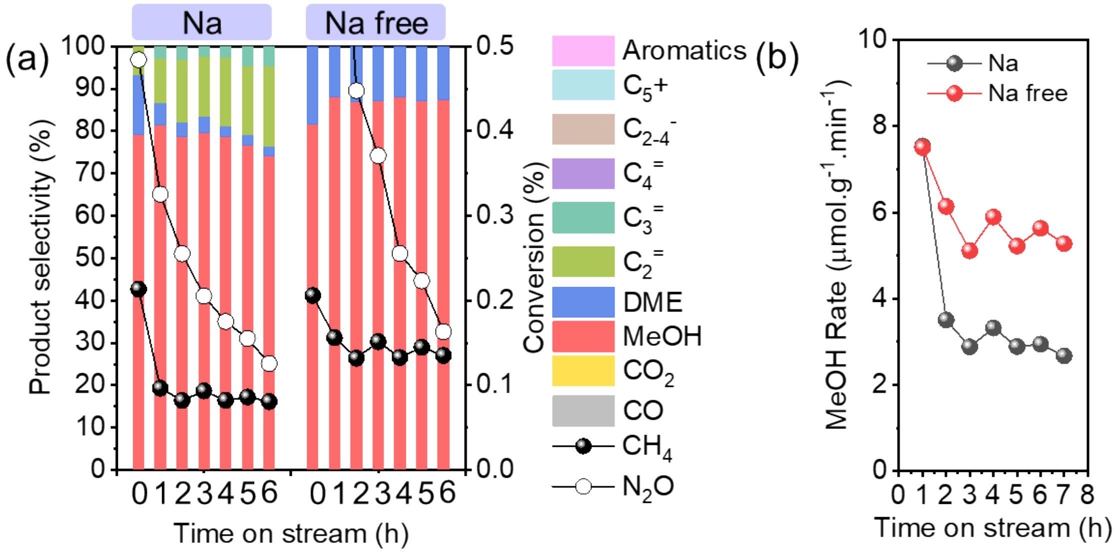

Figure S19. Compare (a) conversion and selectivity, and (b) methanol formation rate of H-type IE-Fe/CHA(Na) and IE-Fe/CHA(Na free) at 250 °C. Reaction conditions: 100 mg catalyst, $CH_4/N_2O/H_2O/Ar$ = 10/10/2/3 ml·min^{-1}, WHSV =15000 ml·g^{-1}·h^{-1}.

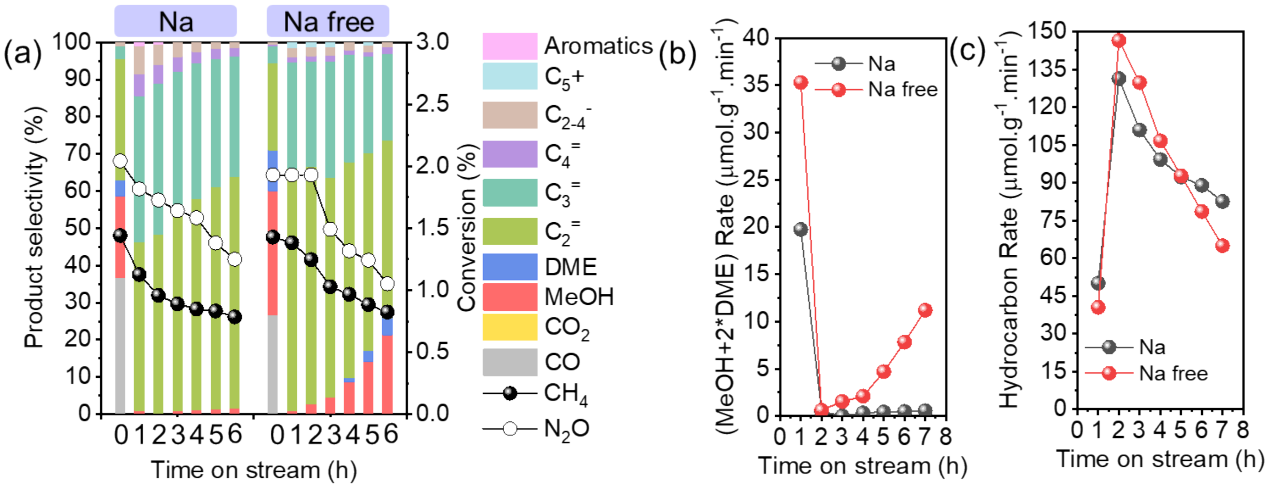

Figure S20. Compare (a) conversion and selectivity, (b) (MeOH+2*DME) formation rate, and (c) hydrocarbon formation rate of H-type IE-Fe/CHA(Na) and IE-Fe/CHA(Na free) at 300 ^oC. Reaction conditions: 100 mg catalyst, $CH_4/N_2O/H_2O/Ar$ = 10/10/2/3 ml·min^{-1}, WHSV =15000 ml·g^{-1}·h^{-1}.

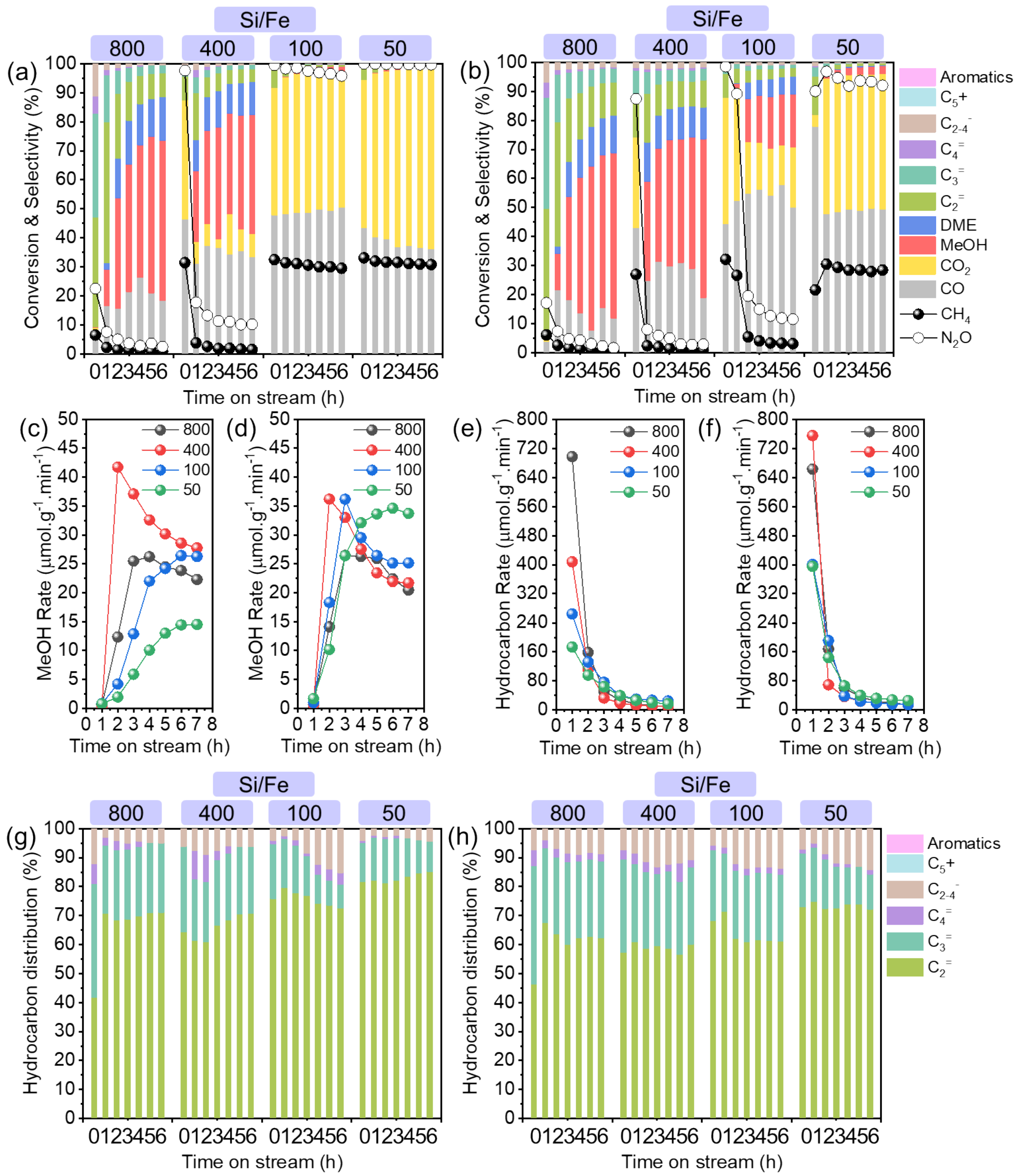

Figure S21. Conversion and selectivity of H-type (a) Fe-CHA(Na)-*x* and (b) Fe-CHA(Na free)-*x* at 350 °C. Compare the methanol formation rate of (c) Fe-CHA(Na)-*x* and (d) Fe-CHA(Na free)-*x*. Compare the hydrocarbon formation rate of (e) Fe-CHA(Na)-*x* and (f) Fe-CHA(Na free)-*x*. Hydrocarbon distribution of H-type (g) Fe-CHA(Na)-*x* and (h) Fe-CHA(Na free)-*x*, where *x* means the Si/Fe ratio in the synthesis gel of 800, 400, 100, and 50. Reaction conditions: 100 mg catalyst, 350 °C, $CH_4/N_2O/H_2O/Ar$ = 10/10/2/3 ml·min^{-1}, WHSV =15000 ml·g^{-1}·h^{-1}.

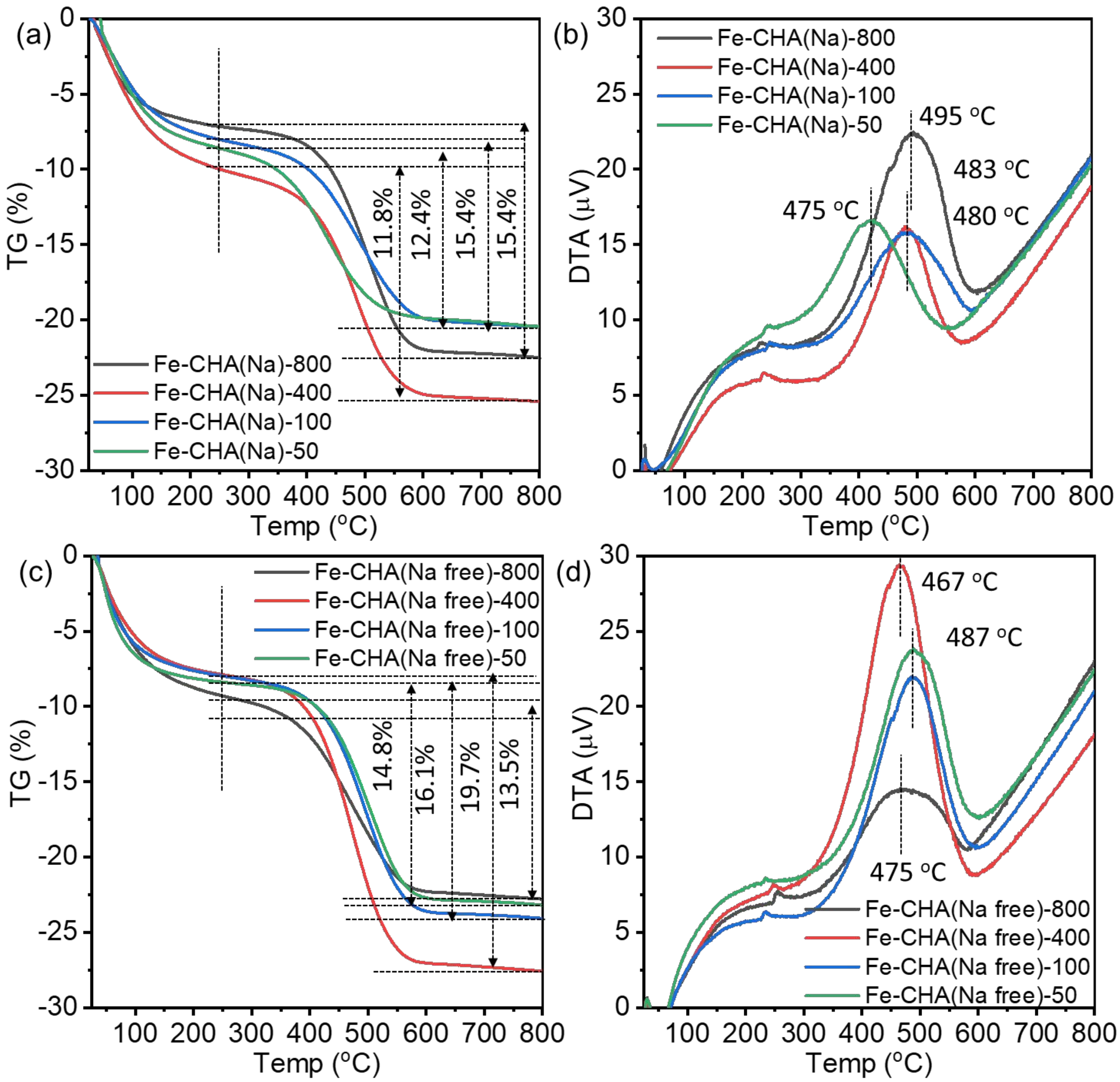

Figure S22. (a) TG and (b) DTA curves of the spend H-type Fe-CHA(Na)-*x* zeolites in methane oxidation reaction at 350 °C, where *x* means the Si/Fe ratio in the synthesis gel of 800, 400, 100, and 50. (c) TG and (d) DTA curves of the spend H-type Fe-CHA(Na free)-*x* zeolites in methane oxidation reaction at 350 °C, where *x* means the Si/Fe ratio in the synthesis gel of 800, 400, 100, and 50.

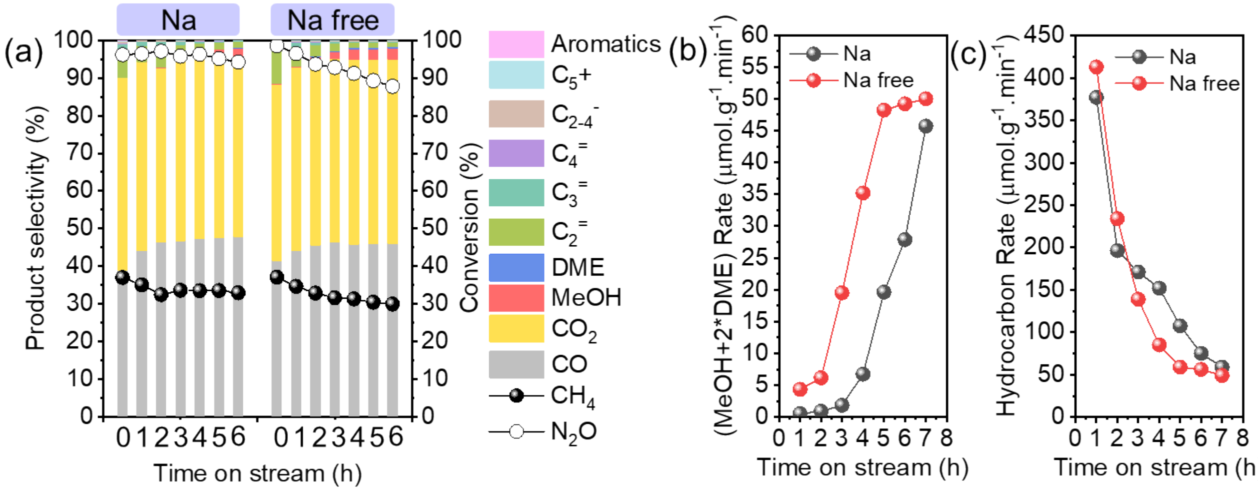

Figure S23. Compare (a) conversion and selectivity, (b) (methanol+2*DME) formation rate, and (c) hydrocarbon formation rate of H-type IE-Fe/CHA(Na) and IE-Fe/CHA(Na free) at 350 ^oC. Reaction conditions: 100 mg catalyst, $CH_4/N_2O/H_2O/Ar$ = 10/10/2/3 $ml \cdot min^{-1}$, WHSV =15000 $ml \cdot g^{-1} \cdot h^{-1}$.

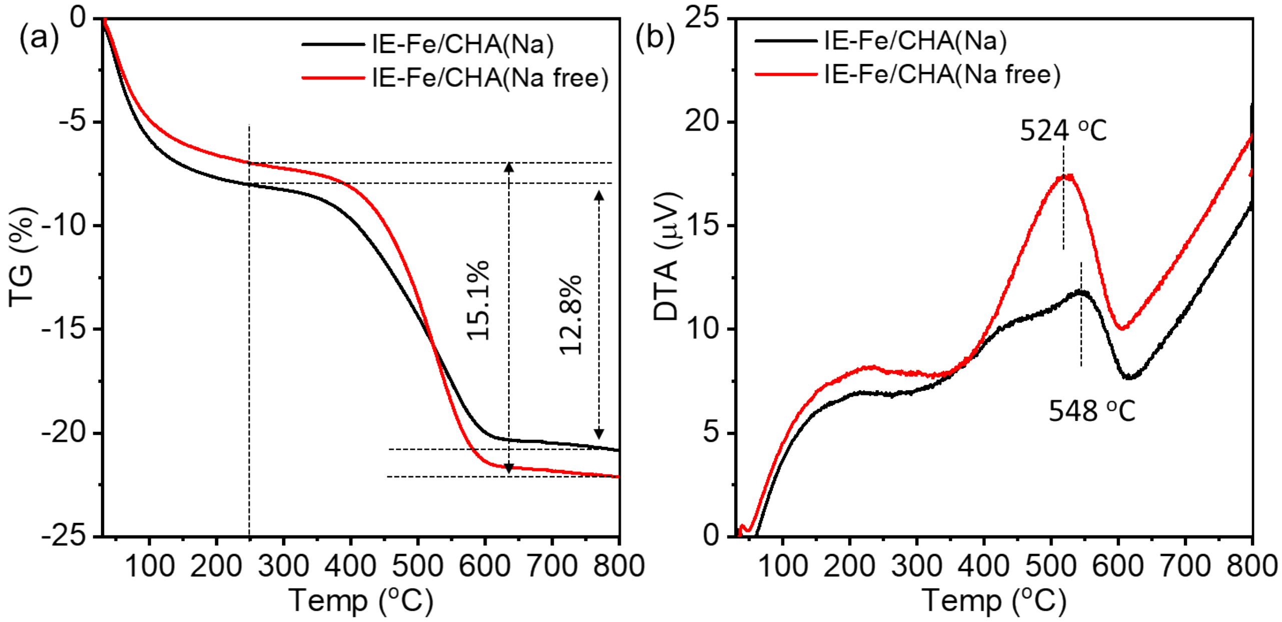

Figure S24. (a) TG and (b) DTA curves of the spend samples in direct oxidation of methane reaction at 350 °C. Reaction conditions: 100 mg catalyst, $CH_4/N_2O/H_2O/Ar$ = 10/10/2/3 ml·min^{-1}, WHSV =15000 ml·g^{-1}·h^{-1}.

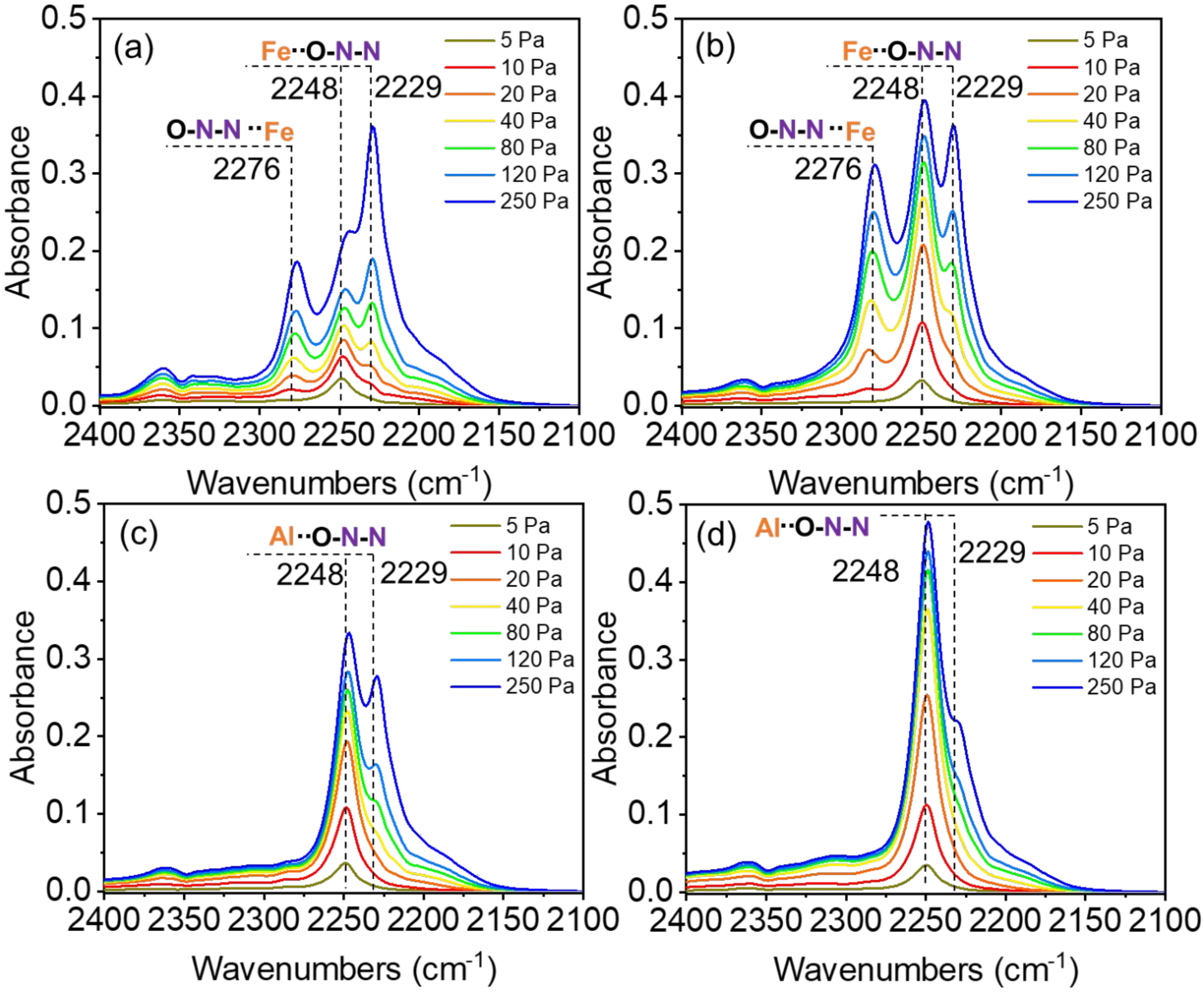

Figure S25. N_2O adsorption FTIR spectra (P_{N2O} = 5−250 Pa) at room temperature over H-type (a) Fe-CHA(Na)-100, (b) Fe-CHA(Na free)-100, (c) CHA(Na), and (d) CHA(Na free) zeolite catalysts after evacuation at 500°C for 1 h.

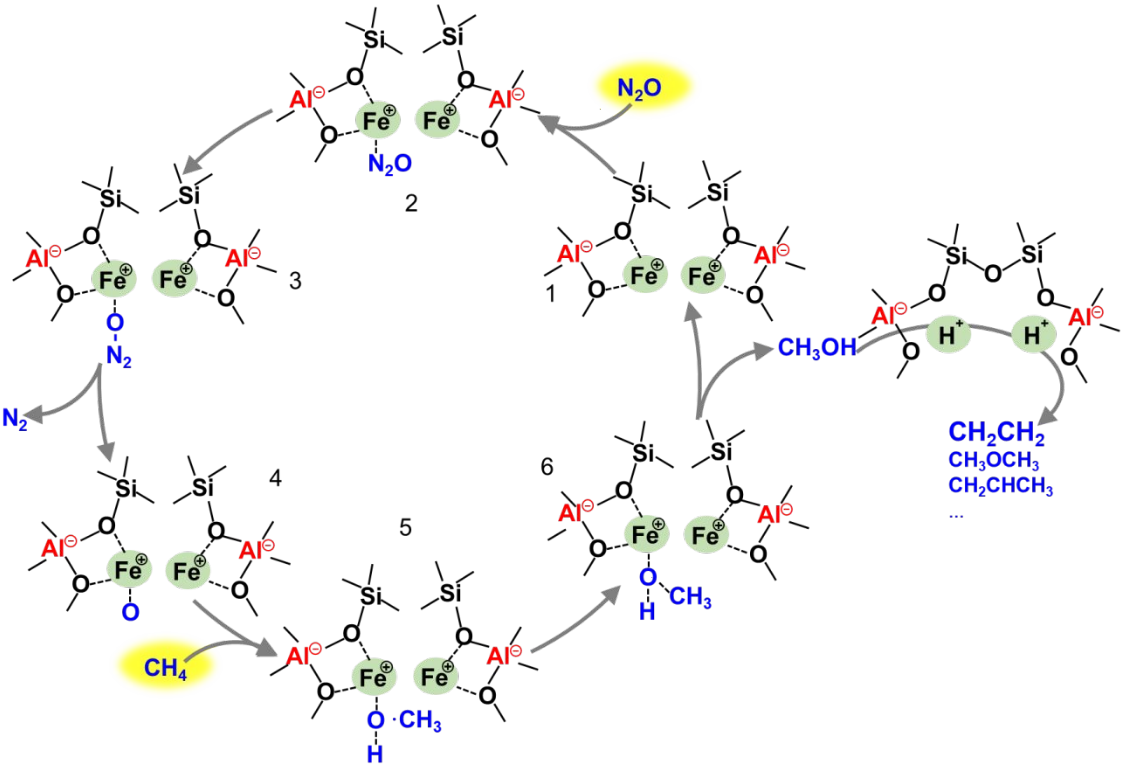

Figure S26. Possible pathway of direct oxidation of CH_4 with N_2O on one of the proximal Fe sites.

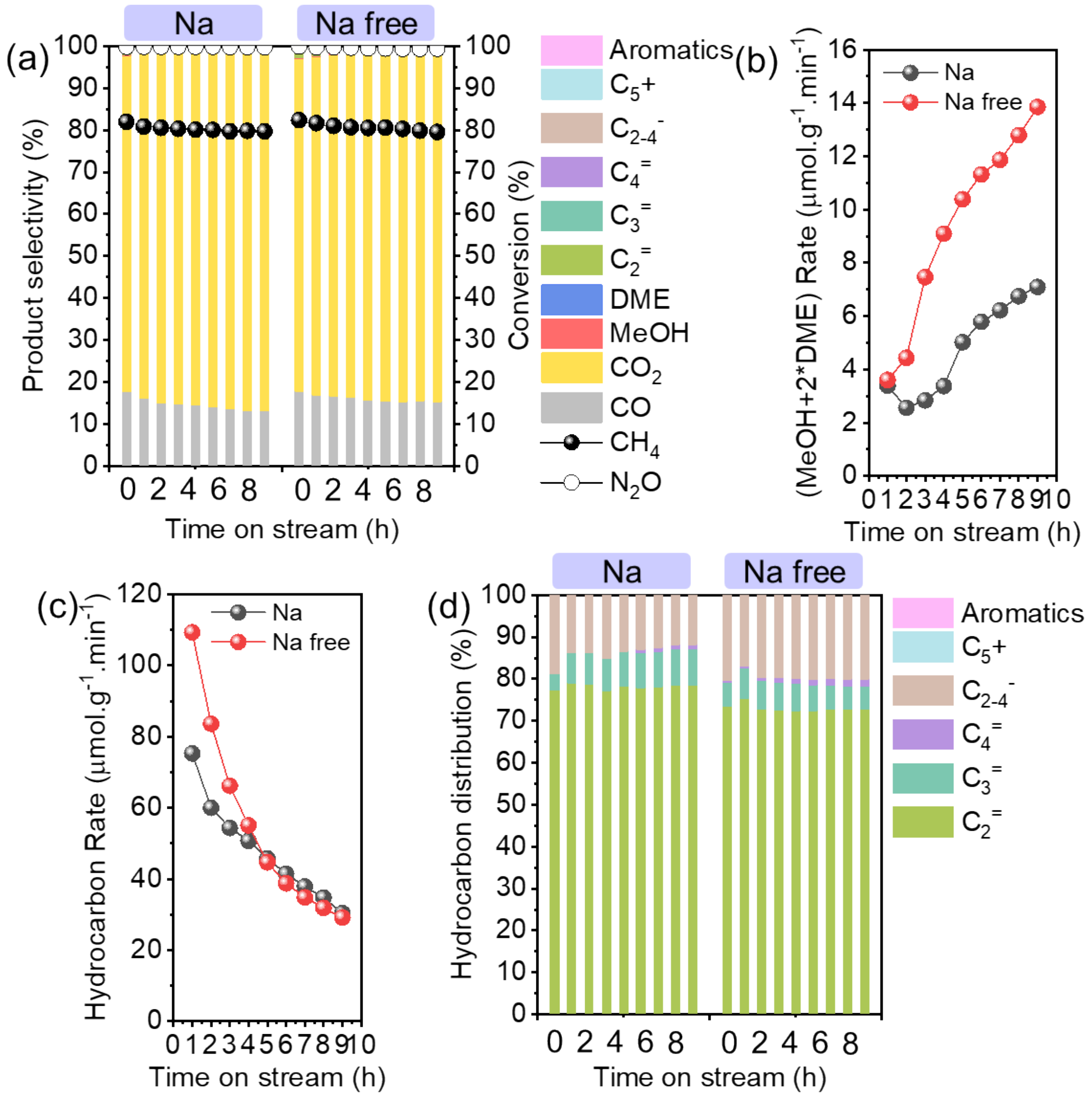

Figure S27. Compare (a) conversion and selectivity, (b) (methanol+2*DME) formation rate, (c) hydrocarbon formation rate, and (d) hydrocarbon distribution of H-type Fe-CHA(Na) and Fe-CHA(Na free) at 350 °C under the insufficient CH_4 condition. Reaction conditions: 100 mg catalyst, $CH_4/N_2O/H_2O/Ar$ = 5/15/2/3 ml·min^{-1}, WHSV =15000 ml·g^{-1}·h^{-1}.

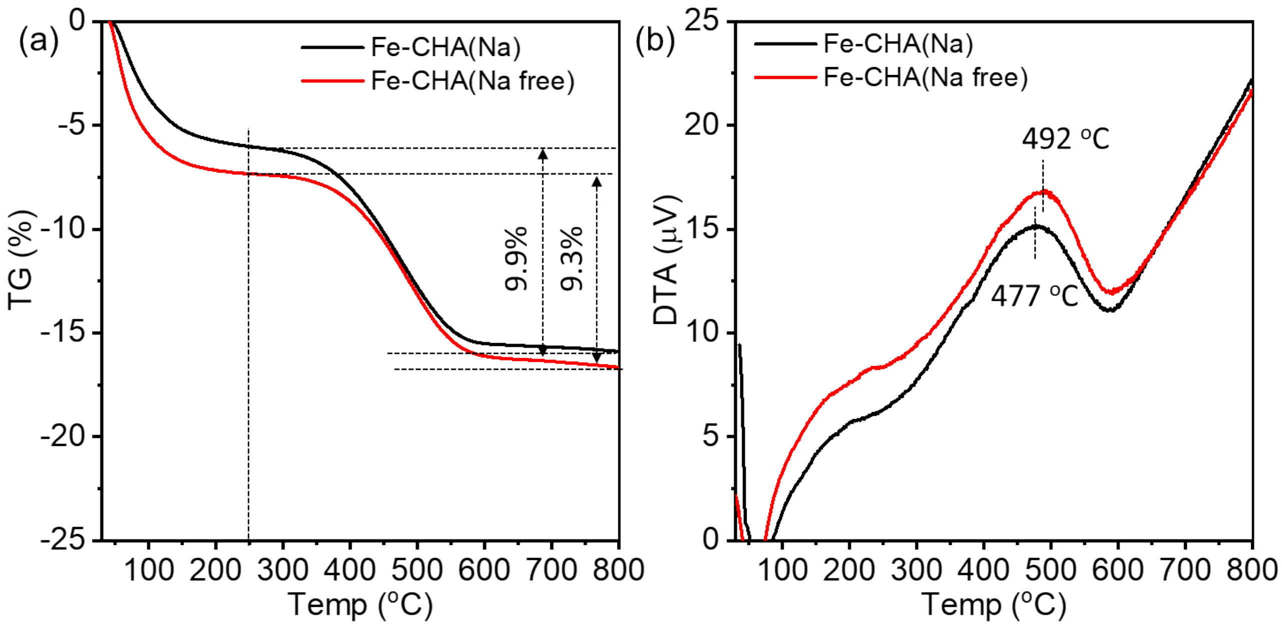

Figure S28. (a) TG and (b) DTA curves of the spent samples in the direct oxidation of methane reaction at 350 °C. Reaction conditions: 100 mg catalyst, $CH_4/N_2O/H_2O/Ar$ = 5/15/2/3 ml·min^{-1}, WHSV =15000 ml·g^{-1}·h^{-1}.

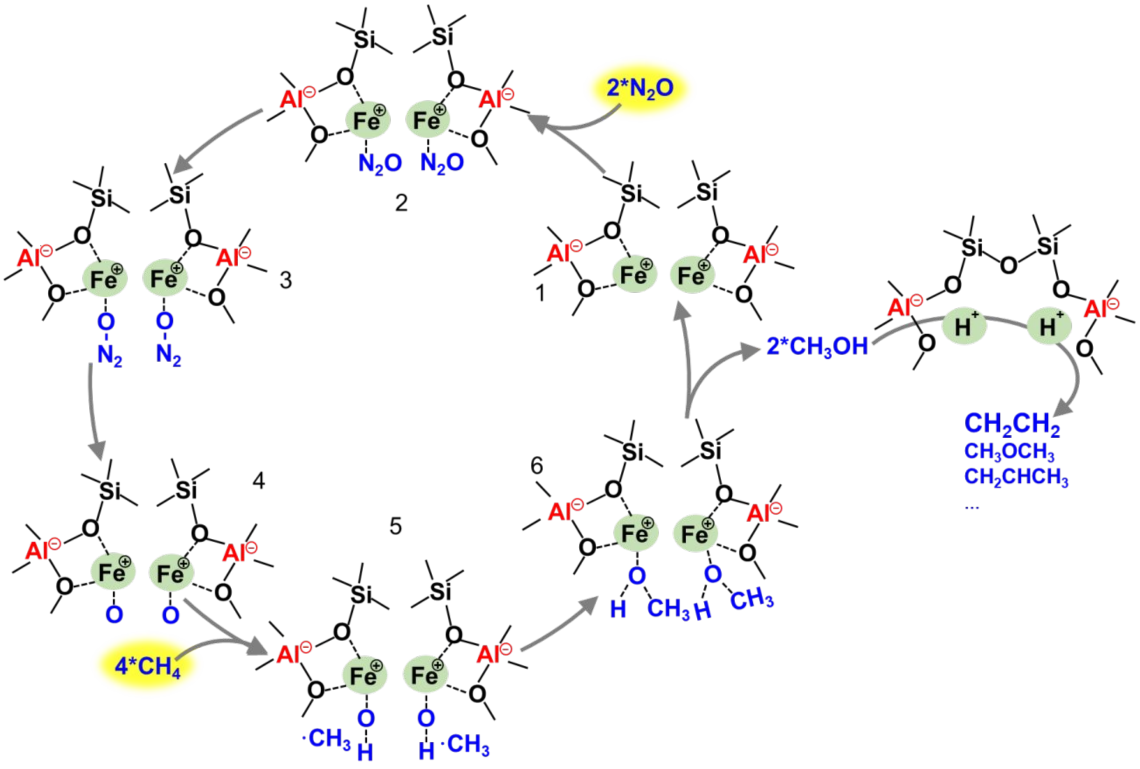

Figure S29. Possible pathway of direct oxidation of CH_4 with N_2O on the proximal Fe sites with sufficient CH_4.

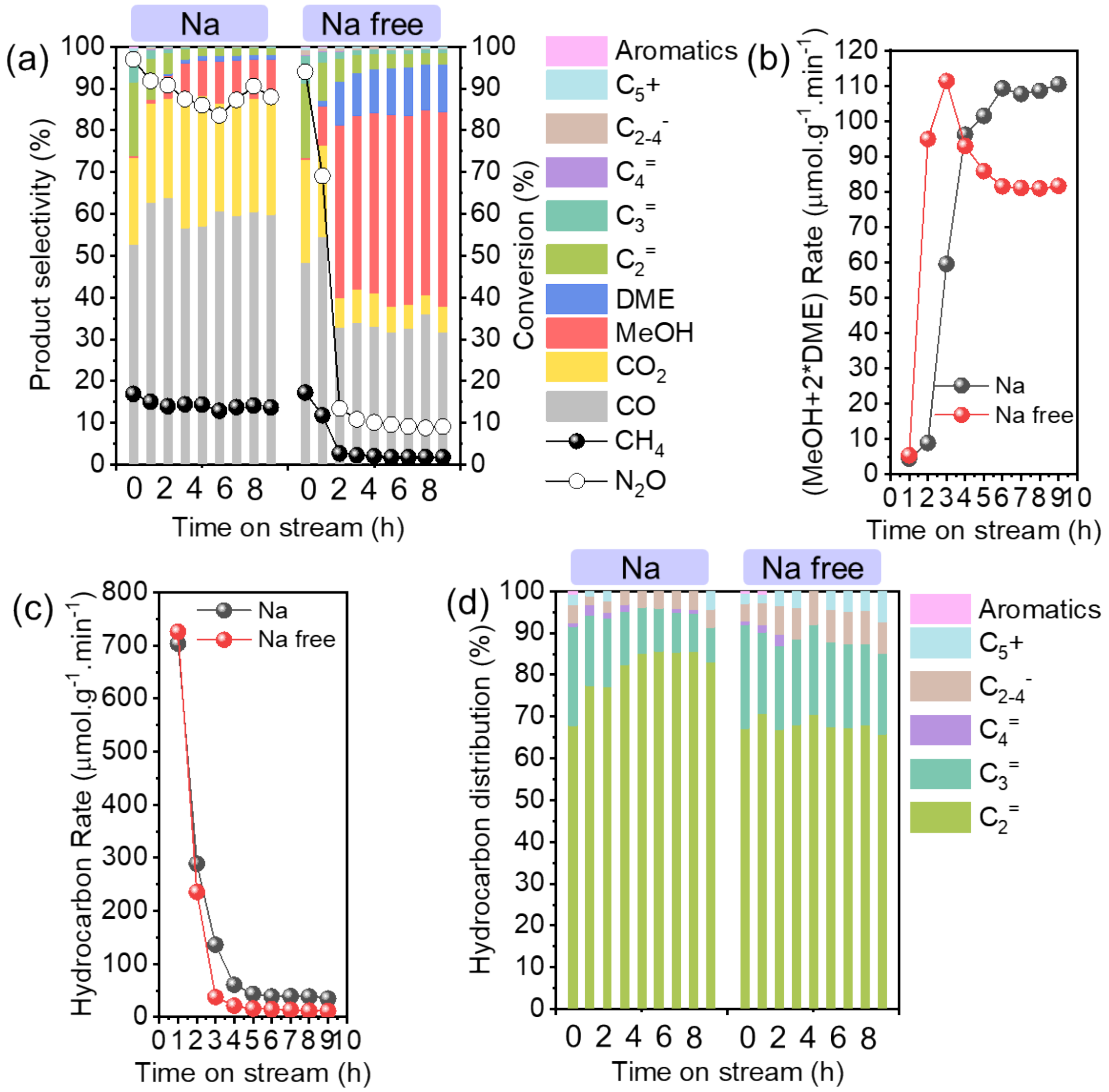

Figure S30. Compare (a) conversion and selectivity, (b) (methanol+2*DME) formation rate, (c) hydrocarbon formation rate, and (d) hydrocarbon distribution of H-type Fe-CHA(Na) and Fe-CHA(Na free) at 350 °C under the sufficient CH_4 condition. Reaction conditions: 100 mg catalyst, $CH_4/N_2O/H_2O/Ar$ = 15/7.5/2/0.5 ml·min^{-1}, WHSV =15000 ml·g^{-1}·h^{-1}.

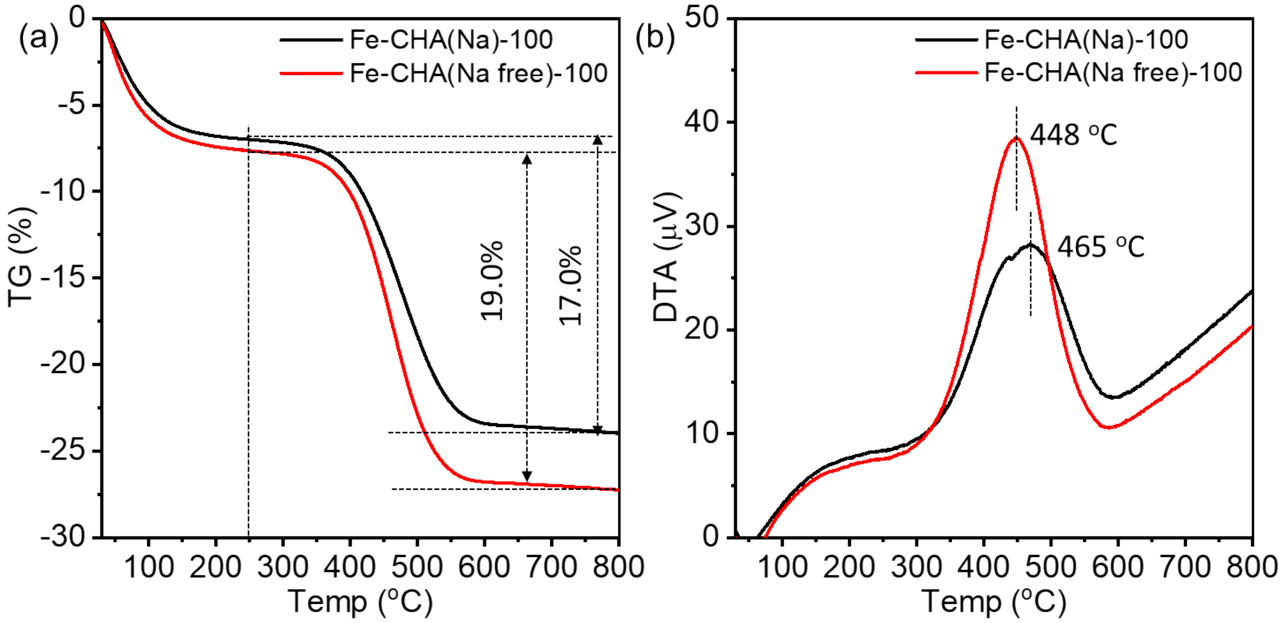

Figure S31. (a) TG and (b) DTA curves of the spend samples in the direct oxidation of methane reaction at 350 °C. Reaction conditions: 100 mg catalyst, $CH_4/N_2O/H_2O/Ar$ = 15/7.5/2/0.5 ml·min^{-1}, WHSV =15000 ml·g^{-1}·h^{-1}.

Table S1. Texture properties of the zeolites measured by N_2 adsorption and desorption.

Sample	$S_{BET}(m^2\cdot g^{-1})$ [a]	$V_{Total}(cm^3\cdot g^{-1})$ [a]	$S_{EXT}(m^2\cdot g^{-1})$ [b]	$V_{Mic}(cm^3\cdot g^{-1})$ [b]
Fe-CHA(Na)-800	790	0.42	30	0.27
Fe-CHA(Na)-400	775	0.39	32	0.27
Fe-CHA(Na)-100	783	0.37	18	0.28
Fe-CHA(Na)-50	729	0.34	8	0.28
Fe-CHA(Na free)-800	823	0.62	101	0.26
Fe-CHA(Na free)-400	874	0.64	103	0.28
Fe-CHA(Na free)-100	776	0.63	88	0.27
Fe-CHA(Na free)-50	738	0.62	65	0.28
IE-Fe/CHA(Na)	689	0.38	15	0.28
IE-Fe/CHA(Na free)	777	0.49	10	0.34

[a] Calculated using the Brunauer–Emmett–Teller (BET) equation on the N_2 adsorption isotherms.
[b] Calculated by the t-plot method based on the adsorption isotherms.

Table S2. Al arrangement of Fe-CHA zeolites determined by fitting ^{29}Si MAS NMR spectra.

Sample	Q^4(0Al)	Q^4(1Al)	Q^3(1OH)	Q^4 (2Al)
	(-120)~(-110) ppm	(-110)~(-100) ppm	(-105)~(-100) ppm	(-100)~(-92) ppm
Fe-CHA(Na)-800	69	25	3	3
Fe-CHA(Na)-400	67	26	4	2
Fe-CHA(Na)-100	69	24	3	4
Fe-CHA(Na)-50	67	24	5	2
Fe-CHA(Na free)-800	72	22	2	3
Fe-CHA(Na free)-400	71	22	6	2
Fe-CHA(Na free)-100	73	22	6	2
Fe-CHA(Na free)-50	69	22	7	2
H-CHA(Na)	77	20	1	2
H-CHA(Na free)	83	12	5	0

Table S3. Chemical composition and density of the Fe-CHA zeolites.

Sample	Chemical Compositions [a]			Al content [b]		Fe content	
	Si/Al	Fe/Al	Fe (wt.%)	Al/unit cell	Al/cage	Fe/unit cell	Fe/cage
Fe-CHA(Na)-800	11.5	0.007	0.05	2.9	1.0	0.02	0.01
Fe-CHA(Na)-400	11.6	0.017	0.11	2.9	1.0	0.05	0.02
Fe-CHA(Na)-100	12.2	0.081	0.54	2.7	0.9	0.22	0.07
Fe-CHA(Na)-50	11.7	0.192	1.31	2.8	0.9	0.54	0.18
Fe-CHA(Na free)-800	12.9	0.011	0.07	2.6	0.9	0.03	0.01
Fe-CHA(Na free)-400	12.7	0.024	0.16	2.6	0.9	0.06	0.02
Fe-CHA(Na free)-100	12.6	0.103	0.67	2.6	0.9	0.27	0.09
Fe-CHA(Na free)-50	12.8	0.188	1.19	2.6	0.9	0.49	0.16

[a] Determined by ICP-AES.
[b] Unit cell of the CHA framework contains 36-T atoms and three CHA cages.[2]

Table S4. Chemical composition and acid amount of the Fe-exchanged CHA zeolites.

Sample	Chemical Compositions [a]				Acid amount (mmol/g) [b]			
	Si/Al	Si/Fe	Fe/Al	Fe (wt.%)	Weak	Medium	Strong	Total
IE-Fe/CHA(Na)	9.3	105	0.089	0.72	0.36	0.33	0.71	1.40
IE-Fe/CHA(Na free)	10.2	100	0.102	0.78	0.23	0.24	0.57	1.04

[a] Determined by ICP-AES.

[b] Determined by NH_3-TPD; the weak, medium, and strong acid amounts were fitted at approximately 150, 300, and 400-450 °C, respectively.

Reference

1. Ravel, B.; Newville, M.; *J. Synchrotron Radiat.* **2005**, 12, 537-541.
2. Bello, E.; Ferri, P.; Nero, M.; Willhammar,T.; Millet,I.; Schütze, F. W.; van Tendeloo, L.; Vennestrøm, P.N.R.; Boronat, M.; Corma, A.; Moliner, M.; NH3-SCR catalysts for heavy-duty diesel vehicles: Preparation of CHA-type zeolites with low-cost templates, *Appl. Catal. B*, **2022**, 303,120928.
